# Bone remodeling stimulated by Wnt-mediated mitophagy regulated extracellular vesicles in subchondral bone contributes to osteoarthritis development

**DOI:** 10.7150/thno.111724

**Published:** 2025-09-21

**Authors:** Yuyuan Gu, Qirong Zhou, Shihao Sheng, Huijian Yang, Dan Huang, Qin Zhang, Hao Zhang, Zijian Cao, Yuanwei Zhang, Zuhao Li, Yingying Jiang, Xiao Chen, Yingying Jing, Chenglong Wang, Hongbo Tan, Ke Xu, Jiacan Su

**Affiliations:** 1 Department of Orthopedics, Xinhua Hospital Affiliated to Shanghai JiaoTong University School of Medicine, Shanghai 200092, P. R. China.; 2 Department of Orthopedics, People's Liberation Army Joint Logistic Support Force 920th Hospital, Kunming City, Yunnan 650032, P. R. China.; 3 Institute of Translational Medicine, Shanghai University, Shanghai 200444, P. R. China.; 4 Organoid Research Center, Shanghai University, Shanghai 200444, P. R. China.; 5 MedEng-X Institutes, Shanghai University, Shanghai 200444, P. R. China.; 6 Department of Orthopedics, Shanghai Zhongye Hospital, Shanghai 200941, P. R. China.; 7 National Center for Orthopedics, Shanghai Sixth People's Hospital, Shanghai, 200233, P. R. China.; 8 Department of Orthopedic Surgery, Shanghai Sixth People's Hospital, Shanghai, 200233, P. R. China.; 9 Sanming Institute of Translational Medicine, Sanming 365004, P. R. China.; 10 Wenzhou Institute of Shanghai University, Wenzhou Key Laboratory of Tissue Regeneration Medical Materials, Wenzhou 325000, P. R. China.

**Keywords:** osteoarthritis, subchondral bone, osteoblast, extracellular vesicles, mitophagy

## Abstract

**Rationale:** Osteoarthritis (OA) is increasingly understood as a disease involving not only cartilage degeneration but also pathological subchondral bone remodeling. The contribution of osteoblast (OB) heterogeneity and their secreted extracellular vesicles (EVs) to this process remains poorly characterized. This study aims to investigate how EVs from distinct OB subtypes modulate subchondral bone remodeling and contribute to OA progression.

**Methods:** OB subtypes representing endothelial (EnOBs), stromal (StOBs), and mineralizing (MinOBs) stages were generated by time-controlled osteogenic induction of BMSCs. EVs were isolated from each OB subtype and characterized by TEM, Western blot, DLS, and miRNA profiling. Functional assays included osteogenic induction, angiogenesis, and cartilage degradation analyses in vitro. RNA-seq and qRT-PCR were used to identify relevant signaling pathways and miRNAs. In vivo effects of EVs were tested in a DMM-induced OA mouse model using intravenous injections, followed by histology, micro-CT, and immunostaining.

**Results:** EVs derived from different OB subtypes exhibited distinct pro-osteogenic, pro-angiogenic, and cartilage-degrading effects. MinOB-derived EVs significantly enhanced osteogenic differentiation and mineralization, correlated with enrichment of calcium phosphate content and specific pro-osteogenic miRNAs. These EVs also carried amorphous calcium phosphate and mitochondrial content, linked to activated mitophagy. Wnt signaling dynamically regulated mitophagy and EV composition, particularly in MinOBs. In vivo, tail vein administration of OB-derived EVs exacerbated subchondral bone sclerosis and cartilage degradation in a time-dependent manner, with MinOB-EVs inducing the most pronounced pathological changes.

**Conclusions:** OB-derived EVs exhibit subtype-dependent regulatory functions in subchondral bone remodeling, mediated by distinct miRNA profiles and mineral cargo shaped by Wnt-regulated mitophagy. These EVs actively participate in OA progression, and their effects vary with disease stage and route of administration. Targeting specific OB subtypes or modulating Wnt-mitophagy signaling may offer novel therapeutic strategies for stage-specific OA intervention.

## Introduction

Osteoarthritis (OA) is the most prevalent form of joint disease worldwide, driven by a multifactorial array of risk factors and significantly impairing the quality of life for millions of individuals globally 1. However, apart from late-stage surgical interventions such as knee joint replacement, current disease-modifying therapies for OA are largely limited to symptom relief, primarily targeting joint pain 2. One of the main reasons for this therapeutic limitation is the insufficient understanding of OA pathogenesis 3, 4.

Although OA has traditionally been viewed as a cartilage-centric disease, it is now increasingly recognized that alterations in the subchondral bone play a critical role in its initiation and progression 5. Recent studies and clinical evidence have demonstrated that the maintenance of articular cartilage integrity is closely linked to appropriate bone remodeling in the subchondral bone 6. In particular, aberrant bone remodeling and disrupted angiogenesis within the subchondral bone have emerged as crucial contributors to OA progression. During the early stages of OA, subchondral bone is characterized predominantly by increased bone resorption, whereas in the later stages, there is a marked shift towards subchondral sclerosis, driven by elevated osteoblastic activity and excessive bone formation 7, 8. Osteoblasts (OBs), which are mesenchymal lineage cells essential for bone formation and the maintenance of skeletal architecture, act as key regulators of this process. Single-cell transcriptomic analyses have revealed diverse OB subtypes with distinct gene signatures and functions. Based on our previous work, OBs can be broadly categorized into three subtypes: endothelial osteoblasts (EnOBs), stromal osteoblasts (StOBs), and mineralizing osteoblasts (MinOBs). EnOBs are enriched in angiogenesis-related signaling pathways and express markers such as VEGF, indicating their involvement in early osteogenesis and vascular coupling. In contrast, StOBs exhibit high expression of extracellular matrix-related genes and are associated with fibrosis-like responses observed in OA. MinOBs are characterized by the expression of mineralization-associated markers and Wnt pathway inhibitors such as WIF1, suggesting a potential role in abnormal mineral deposition and subchondral sclerosis. These subtypes represent a continuous differentiation trajectory, and their relative proportions shift dynamically during different stages of OA, implying that they may play temporally distinct roles in regulating subchondral bone remodeling.

Given that osteoblasts not only perform direct cellular functions but also modulate their microenvironment through secreted extracellular vesicles (EVs), it is plausible that the heterogeneity among OB subtypes is reflected in the composition and function of their EVs. EVs as membrane-enclosed vesicles involved in the transfer of proteins, nucleic acids, and lipids between cells, thereby modulating a wide range of physiological and pathological processes. Importantly, EVs derived from osteoblasts at various stages of differentiation have been shown to possess distinct molecular cargos and functional characteristics 9, suggesting that their biological effects may be stage-dependent. In line with this, recent studies have demonstrated that exosomes secreted from osteoblasts residing in sclerotic subchondral bone regions can contribute to cartilage degeneration in OA models 10-12. These findings collectively suggest that the functional heterogeneity of OB-derived EVs may reflect the underlying diversity of OB subtypes, raising the hypothesis that specific subpopulations of osteoblasts regulate subchondral bone remodeling through the secretion of functionally distinct EVs. While OB-derived EVs are increasingly recognized as important mediators of intercellular communication within the bone-cartilage unit, the mechanisms by which different OB subtypes exert their effects through EVs remain largely undefined. Building upon this foundation, the present study focuses on elucidating the biological functions of EVs secreted by distinct OB subtypes, with the aim of clarifying their contribution to subchondral microenvironmental remodeling during OA progression.

In this study, we characterized the heterogeneity of EVs derived from previously identified OB subtypes. We then analyzed their molecular cargo to uncover potential determinants of functional diversity. Furthermore, we examined how EVs from different OB subtypes influence OA progression by modulating the subchondral bone microenvironment. Collectively, our findings provide new insights into the role of OB-derived EVs in OA pathophysiology and suggest that targeting stage-specific osteoblast subtypes may offer a promising therapeutic strategy.

## Material And Methods

### Cells

Mouse bone marrow-derived mesenchymal stromal cells (BMSC) and the osteoblast precursor cell line (MC3T3-E1) (all from Oricell) were maintained in α-MEM (Corning), while human umbilical vein endothelial cells (HUVECs) (Oricell) were cultured in DMEM (Corning). Chondrocytes were isolated from neonatal Bcl/C57 mouse articular cartilage via enzymatic digestion with type II collagenase (Worthington Biochemical) for 12 h, a well-established method preserving chondrocyte phenotype 13, 14. After digestion, cells were plated in DMEM/F-12 supplemented with 10% FBS and antibiotics (100 U/mL penicillin-streptomycin, Gibco) and cultured at 37 °C with 5% CO_2_. Chondrocyte morphology and phenotype, confirmed by typical cobblestone appearance and expression of Col-2A1 and ACAN at day 2, indicated successful isolation and maintenance.

Osteogenic differentiation was induced in approximately 80% confluent BMSCs and MC3T3-E1 cells using medium containing 0.2 mM ascorbic acid, 10 nM dexamethasone, and 10 mM β-glycerophosphate.

Collection of conditioned culture media (CM): BMSC were cultured with osteoblast differentiation medium for 1 day, 5 day and 9 day respectively, which represent EnOBs, StOBs and MinOBs. Following an initial period of incubation and removing the supernatant from the culture. Then, replaced this with a fresh batch of DMEM without FBS and continued the incubation for an additional 24 h at a temperature of 37 °C. The supernatant involved a centrifugation step at 1000 rpm for 5 min. Once we had the cell-free supernatant, we added FBS to it. This process resulted in the creation of three distinct types of conditioned media (CM): EnOB-CM, StOB-CM, and MinOB-CM. All conditioned culture media were temporarily stored at 4 ℃ for subsequent use or at -80 °C for prolonged storage. Negative controls comprised the culture medium from BMSC, and the vehicle consisted of pure α-MEM.

### Angiogenesis assay, Scratch wound assay and Transwell migration assay

HUVECs were exposed to the CM for 30 min before initiating the angiogenesis assay. Matrigel (BD, #356234) was applied to cover the bottom of a 96-well plate (50 μL/well) and polymerized for 30 min. Subsequently, 1 × 10^4^ HUVECs were seeded into each well, and after a 6-hour incubation, they were labeled with Calcein-AM (Beyotime).

Transwell migration assay: HUVECs (2 × 10^4^) suspended in FBS-free DMEM were gently seeded into the upper chamber of a transwell insert (#353097, FALCON) and cultured with CM for 48 h. Migrated cells were fixed with 4% paraformaldehyde for 10 min, stained with 0.03% crystal violet for 30 min, rinsed with PBS, air-dried, and imaged.

Scratch wound assay: HUVECs (2 × 10^5^) were plated in 12-cells plate and scratched with a sterile pipette tip to create a wound. After 48 h of incubation with CM, wound closure was photographed and quantified. Wound healing percentage was calculated using the formula: [(A_0_/A_48_)/A_0_] × 100%, where A₀ is the initial wound area and A₄₈ is the wound area after 48 h.

### Isolation of EVs

The conditioned media derived from different OB subtypes were collected and EVs were isolated using a sequential differential centrifugation approach, as previously described 15. Given the technical difficulties in precisely distinguishing microvesicles from exosomes based on size and biogenesis alone, all isolated vesicular structures are uniformly referred to as EVs throughout this study.

### Cell viability assay

BMSCs were seeded into 96-well plates at a density of 6 × 10^3^ cells/well and allowed to adhere for 24 h. Subsequently, EVs were administered to the culture medium at concentrations of 1, 1.25, 2, and 2.5 mg/mL to assess their effects on BMSCs viability 16.(Figure S3a) Following an additional 24 h incubation, cell viability was determined using the Cell Counting Kit-8 (CCK-8; Dojindo) in accordance with the manufacturer's instructions.

### Incubation of EVs

EV concentrations were determined by total protein content using a bicinchoninic acid (BCA) assay. EVs were added to complete osteogenic medium at 1.25 mg/mL and incubated with BMSCs for 24 h. After pre-treatment, cells were cultured in osteogenic induction medium for varying durations depending on the assay. For gene and protein expression analysis, cells were harvested on day 3. Alkaline phosphatase (ALP) and Alizarin red staining (ARS) staining were performed on days 5 and 9 to evaluate early and late mineralization, respectively 16.

For cellular uptake, EVs were labeled with DiD (Beyotime) and incubated with BMSCs on confocal dishes for 2 h. Actin filaments were stained with 10 μM F-actin probe (Servicebio), and nuclei with DAPI (Beyotime). Samples were washed with PBS and imaged using a laser scanning confocal microscope (FV3000, Olympus, Japan).

### Western blot

Cell lysates and EV proteins were separated by SDS-PAGE and transferred onto PVDF membranes. After blocking (Epizyme, Shanghai), membranes were incubated overnight at 4 °C with primary antibodies to detect target proteins, including anti-RUNX2 (Cell Signaling, #12556), Anti-Sp7/Osterix (Abcam, #209484), BMP2 (Abcam, #284387), rabbit polyclonal anti-mouse Osteocalcin (Abcam, #93876), Col-2 (Proteintech, #28459-1-AP), CD63 (Abcam, #217345), CD9 (Abcam, #307085), Calnexin (Abcam, #22595), TSG101 (Abcam, #125011), COX IV (Abcam, #202554), SOX9 (Proteintech, #67439-1-Ig), TOMM20 (Abcam, #186735), Wnt3a (Abcam, #219412), Wnt7a (Abcam, #100792), beta-catenin (Cell Signaling, #8480), LC3b (Cell Signaling, #2775), PINK1 (Abcam, #216144), SQSTM1/p62 (Abcam, #109012), PARK2/Parkin (Proteintech, #14060-1-AP), MMP13 (Pproteintech, #18165-1-AP), and GAPDH (Abcam, #181603). Following primary antibody incubation, membranes were washed with TBST and then treated with HRP-conjugated secondary antibodies (Abcam) for 1.5 h at room temperature.

### ALP staining, ARS and Alcian Blue staining

Cells were fixed with 4% paraformaldehyde for 10 min and rinsed with distilled water. For mineralization analysis, ARS (0.2%, Solarbio) was used to stain calcium deposits, and ALP activity was assessed with the ALP Color Development Kit (Beyotime). Mineralized nodules were imaged via a BioTek microplate reader. Quantification was performed by solubilizing ARS-stained cells in 10% cetylpyridinium chloride and measuring absorbance at 562 nm. ALP activity was evaluated using a colorimetric assay kit (Beyotime) per the manufacturer's instructions.

For Alcian Blue staining, chondrocytes pretreated with CM for 24 h were fixed and stained with Alcian Blue (Sigma-Aldrich) to detect glycosaminoglycans, followed by imaging using a microplate reader.

### Transmission electron microscopy (TEM) and Dynamic Light Scattering (DLS) analyze

EVs were placed on carbon-coated copper grids, negatively stained with 2% uranyl acetate, and imaged using a JEM-1400 transmission electron microscope (JEOL, Japan) at 80 kV. For ultrastructural analysis of mineralization and mitophagy, cells were fixed with glutaraldehyde, post-fixed in osmium tetroxide, dehydrated through graded ethanol, embedded in epoxy resin, and examined using a JEM-2100 TEM at 120 kV. Selected Area Electron Diffraction (SAED) was performed on electron-dense and collagen-rich regions to assess crystallinity (five areas per sample). Elemental composition of mitochondria and EVs was analyzed by Energy Dispersive X-ray Spectroscopy (EDX). Both SAED and EDX were performed without staining to preserve ultrastructure. EV size distribution was analyzed by dynamic light scattering (DLS) using a Zetasizer Nano ZSP (Malvern Instruments, UK).

### Immunofluorescence staining

Mitochondrial function was assessed using two fluorescent probes: Mito-Tracker Green for mitochondrial mass and TMRM for membrane potential. Cells were incubated with 10 μM Mito-Tracker Green and 10 μM Lyso-Tracker at 37 °C for 15 min, followed by DAPI nuclear staining for 5 min. After PBS washing, fluorescence imaging was performed using an FV3000 confocal microscope (Olympus, Japan).

For flow cytometric assessment, Mito-Tracker Green and DiD-labeled EVs (Beyotime) were analyzed to determine mitochondrial content within EVs. The mean fluorescence intensity (MFI) was quantified using FlowJo software to reflect the uptake or presence of mitochondria-associated signals in EVs.

### Fourier-Transform Infrared Analysis

Amorphous calcium phosphate (ACP) was synthesized using a co-precipitation method, in which calcium and phosphate ions were combined in the presence of polyphosphate 17. The structural and bonding properties of ACP were characterized via FTIR analysis using a Nexus-470 spectrometer.

### Quantitative real-time polymerase chain reaction (qRT-PCR)

Total RNA was extracted using TRIzol reagent (Takara), and quantified by measuring absorbance at 260 nm. Quantitative PCR was performed on a QTOWER system (Analytik Jena) with a 20 μL reaction mixture. The cycling conditions were: 95 °C for 3 min, followed by 40 cycles of 95 °C for 10 s, 60 °C for 20 s, and 72 °C for 20 s, ending with a final extension at 72 °C for 20 s. Gene expression levels were normalized to GAPDH using the 2^-ΔΔCt method (Mm99999915_g1).

For miRNA analysis, small RNAs were isolated with the MicroRNA Extraction Kit (J&L Biological, AG21030). Reverse transcription followed the same procedure as for total RNA. Expression of ten selected miRNAs was evaluated via SYBR Green qPCR, normalized to the exogenous spike-in control cel-miR-39-3p.

### RNA sequencing and miRNA sequencing

mRNA and small RNA sequencing were performed by OE Biotech Co., Ltd. (Shanghai, China) using the Illumina HiSeq platform. Total RNA was extracted from various osteoblast subtypes and their derived EVs for library construction, with 1 µg RNA used per sample. RNA purity and concentration were assessed by NanoDrop 2000 (Thermo Scientific), and RNA integrity was confirmed using an Agilent 2100 Bioanalyzer. Libraries were prepared with the VAHTS Universal V6 RNA-seq Kit according to the manufacturer's instructions.

For small RNA sequencing, total RNA was isolated via the mirVana miRNA Isolation Kit (Ambion). Small RNA libraries were then constructed using the Small RNA Library Prep Set for Illumina (NEB#E7330S) following the supplier's protocol, and quality was verified on the Agilent 2100 Bioanalyzer.

Differential expression analysis of genes and miRNAs was conducted using DESeq2, with significance thresholds set at P < 0.05 and fold change > 2. Hierarchical clustering was applied to visualize expression profiles. Functional enrichment analyses including Gene Ontology (GO) and KEGG pathways were performed using R (v3.2.0) and supplemented by Hiplot Pro tools.

### Animal

All animal experiments were performed following ethical standards and approved by the Ethics Committee of Shanghai University (Approval No. ECSHU 2023-101). Destabilization of the medial meniscus (DMM) model, an OA model, was induced in male C57BL/6 mice by surgically excising the medial meniscus of the right knee to mimic progressive joint degeneration. Post-surgery, mice in the treatment group received EVs via two administration routes: weekly intravenous tail vein injections at 30 μg/g and intra-articular injections at 1 μg/kg. Animals were sacrificed 8 weeks after surgery for histological analysis.

### Human

This study with human subjects was approved by the Ethics Committee of Shanghai University (Approval No. ECSHU 2021-146). Written informed consent was obtained from all participants before enrollment.

### Micro-computed tomography (Micro-CT) analysis

High-resolution micro-computed tomography (micro-CT; Skyscan 1176, Bruker) was employed to analyze the joint tissues of male C57BL/6 mice with induced osteoarthritis. Joints were fixed in 4% paraformaldehyde (PFA) for 24 h to preserve tissue structure and remove adjacent muscle. Three-dimensional reconstructions were generated for morphological assessment. Quantitative evaluation of subchondral bone remodeling included bone volume fraction (BV/TV) and subchondral bone plate thickness (SBP.th).

### Microangiography

Vascular and structural assessments in animal models were initiated under anesthesia induced with isoflurane (Sigma-Aldrich). Vascular perfusion was performed via intracardiac injection using a sequential infusion protocol consisting of phosphate-buffered saline (PBS) supplemented with anticoagulants, followed by 4% paraformaldehyde (PFA), and subsequently MicroFil MV-120 (Flow Tech), a radiopaque silicone rubber compound designed to delineate vascular architecture. Following perfusion, the tissues were harvested and subjected to decalcification for a period of three weeks to facilitate downstream imaging and analysis.

### Histology, immunochemistry and immunofluorescence analysis

Mouse knee joints were fixed in 4% paraformaldehyde (PFA), followed by paraffin embedding for histological evaluation. Sagittal sections from the medial knee compartment were stained using Hematoxylin and Eosin (H&E) for general morphology and Safranin O/Fast Green (SO&FG) to highlight cartilage and subchondral bone structures. Osteoarthritic severity was quantified via the Osteoarthritis Research Society International (OARSI) scoring system according to established methods 18. For immunolabeling, immunohistochemistry (IHC) and immunofluorescence (IF) were performed using primary antibodies against CD31 (1:50, GB113151, Servicebio) and Osteocalcin (OCN, 1:100, GB11233, Servicebio), with overnight incubation at 4 °C to ensure specific binding.

To evaluate short-term safety of EV treatment, major organs including heart, liver, spleen, lungs, and kidneys were collected for histopathological assessment. Paraffin sections underwent H&E staining to identify any morphological abnormalities or pathological changes.

### ELISA

To evaluate systemic inflammation, serum levels of key pro-inflammatory cytokines—interleukin-1β (IL-1β), interleukin-6 (IL-6), and tumor necrosis factor-α (TNF-α)—were measured. Blood samples were collected from all experimental groups, and serum was separated for cytokine analysis. Concentrations were determined using ELISA kits (MultiSciences) following the manufacturer's protocols, with optical density measured at 450 nm by a microplate reader.

### Statistical analysis

All data are presented as mean ± SD from at least three independent biological replicates. Statistical analyses were performed using GraphPad Prism 8 and Excel 2016. Differences between two groups were assessed by unpaired Student's t-test. One-way ANOVA was used for comparisons among three or more groups under one factor, while two-way ANOVA evaluated the effects of two factors and their interaction. Tukey's HSD test was applied for post hoc multiple comparisons. Significance was set at p ≤ 0.05, indicated as *p < 0.05, **p < 0.01, ***p < 0.001, and ****p < 0.0001; nonsignificant differences are marked “ns.” In bar graphs, identical letters denote no significant difference. Error bars represent mean ± SD.

## Results

### Osteoblast heterogeneity drives subchondral bone remodeling in osteoarthritis

**Figure 1:**
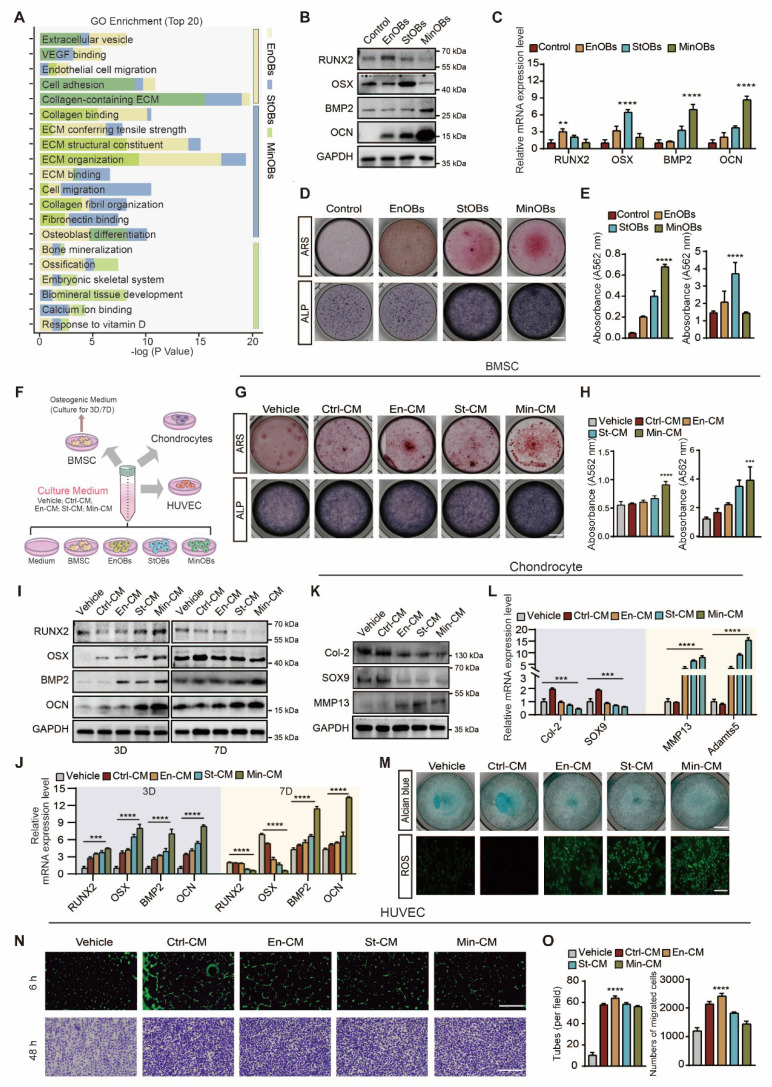
** Identification of OB subtypes and the functions to chondrocytes, BMSC and HUVECs. (A)** GO enrichment analysis of osteoblast subtypes in subchondral bone from OA patients by scRNA-seq. **(B-C)** Western Blot and qRT-PCR analysis validated the osteogenic genes expressed in EnOBs, StOBs and MinOBs.** (D-E)** ARS and ALP staining of OB subtypes and relative quantitative analysis. Scale bar: 200 μm. **(F)** Schematic showing the strategy for chondrocytes, BMSC and HUVECs treated with culture medium (CM) form three OB subtype. **(G-H)** ARS and ALP staining of BMSC after treated with CM for 5 days and 9 days respectively and relative quantitative analysis. Scale bar: 200 μm. **(I-J)** Western blot and qRT-PCR were used to analyze the expression levels of RUNX2, Osterix, BMP-2, and osteocalcin (OCN) in Bone Marrow Stromal Cells (BMSC) treated with Conditioned Media (CM) and subjected to osteogenic induction over periods of 3 and 7 days. **(K)** Western blot analysis of the protein level in chondrocytes treated with CM for 24 h. **(I)** qRT-PCR analysis of the mRNA level of SOX9, Col-2, MMP13 and Adamts5. **(M)** Alcian blue staining of chondrocytes and ROS localization in chondrocytes treated with CM for 24 h. anova: 200 μm; 500 μm. **(N)** The formation of tubes by HUVECs was assessed following a 6-hour incubation on Matrigel. Images of migrating cells were captured through crystalline violet staining. Scale bar: 500 μm.** (O)** Statistical analysis of the numbers of formed tubes and migration rate of HUVECs. Differences in mean values were evaluated for statistical significance using Tukey's HSD multiple comparisons test at a significance level of p ≤ 0.05. The results, depicted graphically with representative images, are based on three independent replicates, and error bars represent the mean ± SD. Means sharing the same letters (ns) are not considered significantly different. The following significance levels were assigned: *p < 0.05, **p < 0.01, ***p < 0.001 and ****p < 0.0001. Quantitative data are presented as mean ± SD.

In the previous study, we identified three OBs subtypes within subchondral bone of OA patients, EnOBs, StOBs and MinOBs, each exhibiting unique regulatory roles in bone remodeling 19. (Figure 1A) Trajectory analysis of single-cell RNA sequencing (scRNA-seq) data revealed a progressive differentiation pathway from EnOBs to MinOBs, with StOBs occupying an intermediate transitional state. (Figure S1A) To investigate whether such OB heterogeneity could be reproduced in vitro, we isolated OB subtypes at different stages of osteogenic differentiation following established protocols 9. (Figure S1B) Subtype-specific markers were subsequently confirmed by molecular analyses. (Figure S1G) EnOBs highly expressed VCAM1 and MGST1, StOBs were characterized by elevated Col1A1 and GFPT2, MinOBs exhibited high expression of IBSP and WIF1. The functional characterization of these osteoblast subtypes was further confirmed by ALP staining, ARS staining, and WB, corroborating their distinct osteogenic phenotypes. (Figure S1C-E) These results highlight the functional diversity among OB subtypes. EnOBs were associated with pathways involved in angiogenesis, StOBs mainly contributed to extracellular matrix (ECM) organization, and MinOBs participated in mineral deposition. These functional attributes are consistent with the scRNA-seq data, supporting the notion that these subtypes play temporally and mechanistically distinct roles in bone remodeling during OA progression.

To further clarify this temporal progression, we examined the expression dynamics of key osteogenic genes across the differentiation timeline. RUNX2, a transcription factor essential for early osteoblast commitment, was upregulated at early-stage (1D) 20. Osterix expression peaked at the mid-stage of osteogenic differentiation (5D), which coincided with ECM maturation and an increase in ALP activity. In contrast, BMP-2 and OCN expression became dominant at the late stage (9D), indicating the initiation of mineralization and matrix accumulation 21, 22. (Figure S1F-G) Based on these findings, we assigned 1D, 5D, and 9D as representative stages corresponding to EnOBs, StOBs, and MinOBs, respectively. Their osteogenic potential at each stage was confirmed through expression of marker genes, (Figure 1B-C) and staining assays for ALP and ARS. (Figure 1D-E)

Aberrant remodeling of subchondral bone in OA is driven by complex interactions among chondrocytes, bone marrow-derived BMSCs, and endothelial cells 8. To evaluate how each OB subtype modulates this microenvironment, we treated BMSCs, chondrocytes, and HUVECs with CM derived from EnOBs, StOBs, or MinOBs. (Figure 1F) Media from MinOBs significantly promoted mineralization in BMSCs (Figure 1G-H) and elevated the expression of early osteogenic genes after 3 days of induction. (Figure 1I-J) Interestingly, the expression of early osteogenic markers, including RUNX2 and Osterix, was downregulated by day 7, consistent with their maturation-dependent decline (Figure S1F-G), suggesting that MinOB facilitates terminal osteogenic differentiation in BMSCs. In chondrocytes, CM from all three OB subtypes led to degradation of the ECM. This was indicated by increased levels of catabolic enzymes MMP13 and Adamts5 (Figure 1K-L), reduced proteoglycan content as shown by Alcian Blue staining, and elevated production of reactive oxygen species (ROS). (Figure 1M) These results suggest a potential catabolic influence of OBs on cartilage integrity. Additionally, CM from EnOBs significantly enhanced HUVEC migration and formation of angiogenic network (Figure 1N-O), which supports their involvement in angiogenesis-associated osteogenesis during the early stages of OA 23. (Figure 1A)

To determine whether these functional characteristics are also present in immortalized cell lines, we conducted parallel experiments using MC3T3-E1-derived OBs. Similar effects on osteogenesis, angiogenesis, and cartilage degradation were observed, (Figure S2A-F) confirming that the functional heterogeneity of OB subtypes is conserved in both primary and established cell models. Altogether, our findings demonstrate that osteoblast subtypes possess distinct, subtype-dependent roles in modulating the cellular microenvironment, thereby contributing to subchondral bone remodeling in OA.

### EVs from osteoblast subtypes exhibiting the ability to regulate subchondral bone remodeling

**Figure 2:**
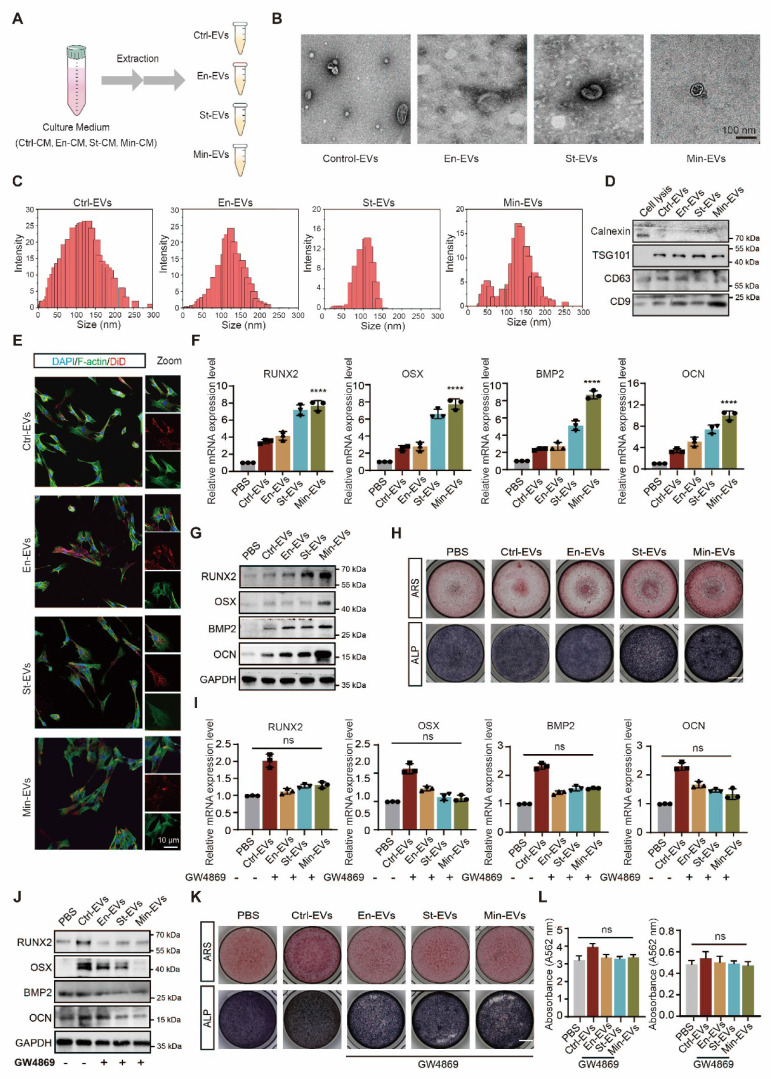
** Osteoblast subtypes-derived extracellular vesicle promote osteogenesis. (A)** A diagrammatic representation outlining the method used for extracting three different types of EVs. **(B)** Representative TEM images of three EVs. Scale bars: 100 nm. **(C)** Analysis of the size distribution of the EVs, as determined by DLS. **(D)** Identification of specific marker proteins present within the EVs. **(E)** EVs were endocytosed into the BMSCs after co-cultured for 6 h located by DID (red) and F-actin (green). Scale bar: 10 μm. **(F-G)** Western blot analysis and qRT-PCR analysis of the expression level of osteogenic markers in BMSC treated with EVs after osteogenic induced for 3 days. **(H)** ARS and ALP staining of BMSC after treated with EVs for 5 days and 9 days respectively. Scale bar: 200 μm.** (I-J)** Western blot analysis and qRT-PCR analysis of the expression level of osteogenic markers in BMSC co-cultured with GW4869-treated EVs after osteogenic induced for 3 days.** (K-L)** ARS and ALP staining of BMSC with GW4869-treated EVs for 5 days and 9 days respectively and relative quantitative analysis. Scale bar: 200 μm.

EVs are increasingly recognized as pivotal mediators of intercellular communication within the subchondral bone microenvironment 24. Previous studies have shown that EVs secreted by OBs can modulate the osteogenic differentiation of BMSCs and modulate chondrocyte metabolism, thereby contributing to OA 25, 26. The role of EVs is now considered central in the pathogenesis of OA 12. To investigate whether distinct OB subtypes regulate bone remodeling through EV-mediated signaling, EVs were isolated from CM using ultracentrifugation techniques. (Figure 2A) TEM revealed that the isolated vesicles exhibited the typical oval morphology characteristic of EVs. (Figure 2B) Nanoparticle tracking analysis showed that EnOB-derived EVs had a mean diameter of approximately 110 nm, while those from StOBs averaged around 100 nm. (Figure 2C) In contrast, EVs derived from MinOBs displayed a broader size distribution, with diameters ranging from 150 to 200 nm. Western blot analysis confirmed the presence of classical exosomal markers, including TSG101, CD63, and CD9, while the endoplasmic reticulum marker Calnexin was absent, indicating the purity of the EV preparations. (Figure 2D) Confocal microscopy futher demonstrated efficient uptake of fluorescently labeled EVs by BMSCs during co-culture, with vesicles accumulating within recipient cells. (Figure 2E)

To confirm the pro-osteogenic potential of OB-derived EVs, various concentrations were first tested to determine the optimal dosage with minimal cytotoxicity. (Figure S3A) Gene expression analysis of osteogenic markers revealed that EVs derived from StOBs and MinOBs significantly promoted osteogenesis. (Figure 2F-G) Notably, EnOB-derived EVs (En-EVs) also showed greater osteogenic potential compared to BMSC-derived EVs (Ctrl-EVs). Furthermore, ALP and ARS staining indicated that MinOB-derived EVs (Min-EVs) induced more robust calcium nodule formation than En-EVs and StOB-derived EVs (St-EVs). (Figure 2H) To confirm that these effects were specifically mediated by EVs, GW4869, a neutral sphingomyelinase inhibitor, was used to suppress EV secretion in OB subtypes. This treatment markedly reduced EV production (Figure S3B) and abolished their osteoinductive effects, as evidenced by decreased ALP activity, reduced mineral deposition, and downregulation of osteogenic markers expression. (Figure 2I-L) These findings confirm that EVs derived from OB subtypes differentially regulate osteogenesis and preserve the functional heterogeneity inherent to their cellular origin, thereby influencing interactions within the subchondral bone microenvironment. Through the regulatory actions of OB-derived EVs, alterations in bone remodeling turnover may accelerate the shift toward bone formation during OA progression 19. In addition to their osteogenic functions, EnOB-derived EVs significantly enhanced the migratory capacity of HUVECs, suggesting their involvement in angiogenic processes. (Figure S2F-G) Moreover, OB-derived EVs exerted pronounced regulatory effects on chondrocytes. All three EV types significantly upregulated the expression of catabolic enzymes MMP13 and Adamts5 (p < 0.05), while simultaneously downregulating the anabolic markers SOX9 and Col-2 (p < 0.01). (Figure S2E) These observations are consistent with previous reports indicating that osteogenesis-angiogenesis coupling is enhanced in the subchondral bone of OA patients, contributing to disease progression.

### miRNAs in EVs are responsible for contributions of OA subchondral bone remodeling

**Figure 3:**
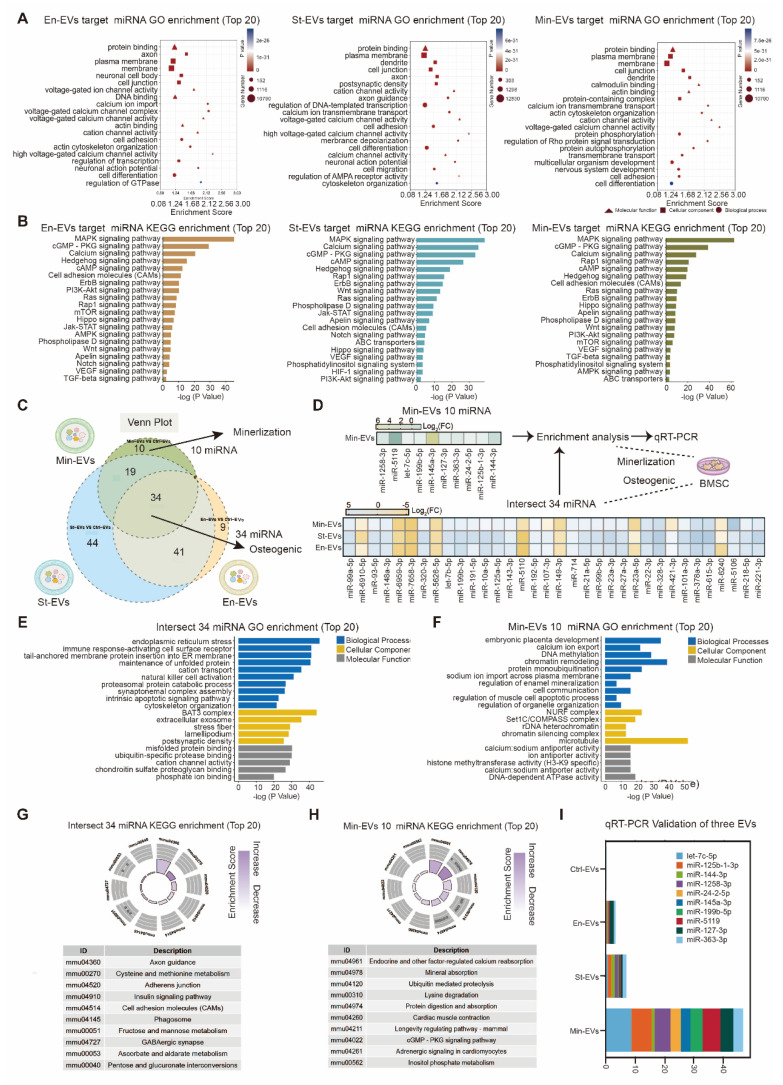
** Functions of miRNAs in osteoblasts subtypes-derived EVs. (A)** GO analysis of pathways and biological processes for differentially expressed miRNAs in three EVs.** (B)** KEGG analysis of pathways and biological processes for differentially expressed miRNAs in three EVs. **(C)** Venn analysis between differentially expressed miRNAs in three EVs. **(D)** Heatmaps for intersect 34 miRNAs in three EVs and unique 10 miRNAs in Min-EVs. **(E)** GO enrichment analysis suggests potential functions for the target genes of the 34 intersecting miRNAs. **(F)** GO enrichment analysis indicates possible functions of target genes associated with 10 miRNAs found in Min-EVs. **(G)** Analysis of KEGG pathways reveals the top 10 pathways linked to the target genes of the 34 intersecting miRNAs. **(H)** KEGG pathway analysis identifies the top 10 pathways associated with the target genes of 10 miRNAs in Min-EVs. **(I)** Bar plot showed the mRNA expression level 10 miRNAs in Min-EVs.

miRNAs carried by EVs serve as critical regulators of intercellular communication within the subchondral bone microenvironment 27. To determine whether miRNAs mediate the pro-osteogenic effects of OB subtype-derived EVs, small RNA sequencing was performed on EVs from the three OB subtypes. The analysis revealed a substantial number of differentially expressed miRNAs across the three EV populations (Figure S4A-C) Notably, miRNA profiles of St-EVs exhibited greater clustering, implying broader functional diversity. To explore the functional implications of these miRNAs, we predicted their target genes using TargetScan database. GO enrichment analysis showed that miRNAs in En-EVs were primarily associated with cell differentiation-related processes. In contrast, St-EVs were enriched in pathways related to cell adhesion, while miRNAs in Min-EVs were associated with the negative regulation of cell differentiation. (Figure 3A) Further KEGG pathway analysis of the differentially expressed miRNAs indicated enrichment in protein binding, MAPK signaling, and calcium signaling pathways, highlighting their potential role in modulating key molecular processes within the subchondral bone niche. (Figure 3B) The enrichment of calcium signaling pathways may be linked to elevated mitochondrial calcium levels in chondrocytes, which can lead to mitochondrial dysfunction and cartilage calcification, thereby exacerbating OA progression 28, 29. Moreover, activation of the MAPK pathway has been implicated in promoting pro-inflammatory responses, oxidative stress, and ECM degradation in articular cartilage 30. These findings suggest that OB-derived EVs may simultaneously disrupt cartilage homeostasis and regulate bone remodeling.

To further identify shared miRNAs potentially contributing to osteogenesis, we performed a Venn analysis of differentially expressed miRNAs across the three EV groups. This analysis revealed 34 intersecting miRNAs, including 26 that were upregulated and 8 that were downregulated. (Figure 3C) GO analysis of these miRNAs and their predicted target genes indicated enrichment in ECM-related functions, such as matrix composition, turnover, and signaling, with phosphate ion binding emerging as the most prominent functional category. (Figure 3E) KEGG pathway analysis also predicted enrichment in axon guidance, a process potentially contributing to enhanced nerve growth and mechanical hyperalgesia observed in OA-associated pain 28. (Figure 3G) Collectively, these findings suggest that miRNAs carried by OB-derived EVs play pivotal roles in ECM organization and calcium/phosphate metabolism during bone remodeling.

Given the robust mineralization capacity of Min-EVs observed in earlier experiments, we next focused on their specific miRNA cargo. Among the differentially expressed miRNAs in Min-EVs, ten were identified, nine of which were significantly upregulated. (Figure 3D) Based on existing literature, we summarized the putative biological functions of these miRNAs in Table 1. For instance, miR-497 has been shown to enhance osteogenic differentiation by targeting Smad7 and activating BMP signaling, thereby promoting matrix mineralization 31. Functional enrichment analysis further revealed that these ten miRNAs are involved in embryonic development and ATP binding, suggesting their roles in supporting growth, differentiation, and cellular metabolism. (Figure 3F) These results are consistent with our earlier findings, supporting the notion that Min-EVs possess enhanced osteogenic and mineralizing potential through their regulation of calcium and phosphate deposition. (Figure 3G) Quantitative real-time PCR confirmed the elevated expression of these ten miRNAs specifically in MinOB-derived EVs, (Figure 3I) establishing them as potential molecular signatures associated with the highly osteogenic MinOB subtype.

### Amorphous calcium phosphate released in heterogeneous EVs driving subchondral bone remodeling

**Figure 4:**
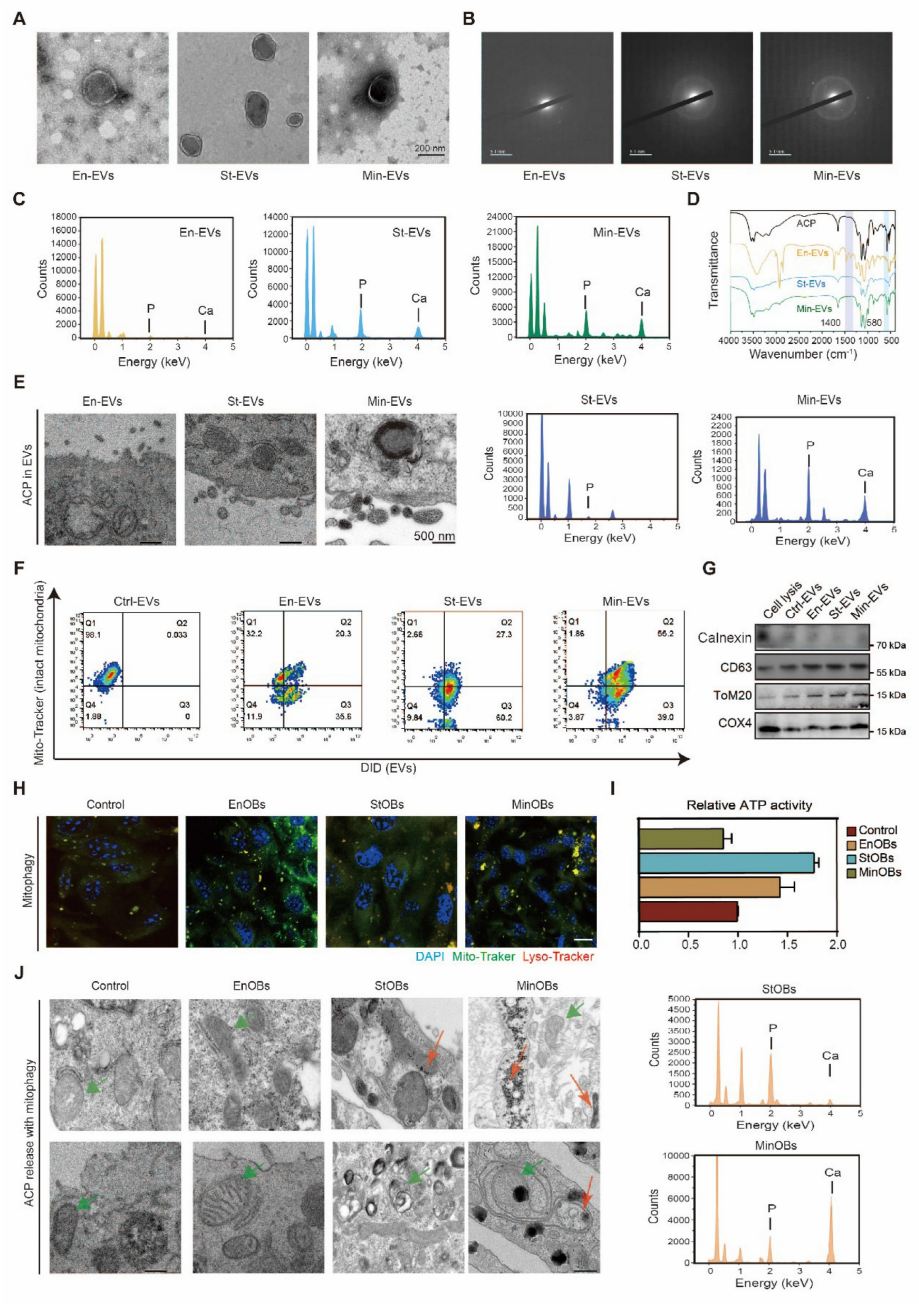
** Intramitochondrial electron-dense amorphous calcium phosphate (ACP) granules contained in EVs. (A)** TEM images show electron-dense granules in EVs. **(B)** SAED revealed the amorphous nature of granules. **(C)** Elemental mapping of granules in EVs demonstrated the presence of calcium and phosphorus (bar: 500 nm). **(D)** FT-IR analysis of ACP and three EVs. **(E)** TEM images show electron-dense granules in OB subtypes released EVs.** (F)** Flow cytometric analysis of Mitotracker Green-labeled mitochondria and DID-labeled cell membrane in EVs. **(G)** Western blotting analysis of mitochondrial maker proteins in EVs. **(H)** Confocal microscopy images depict various OBs subtypes marked with Mito-tracker Green (green) for mitochondria, Lyso-tracker Red (red) for lysosomes, and DAPI (blue) for nuclei. (n = 3). Scale bar: 10 μm. **(I)** ATP activity in three OB subtypes showed the activation of mitochondria.** (J)** High-magnification TEM images displayed intramitochondrial ACP granules (red arrow) within mitochondria (green arrow), and EDX elemental mapping confirmed the presence of calcium and phosphorus (bar: 1/50 nm).

Within developing bone, EVs play an essential role in delivering pro-osteogenic factors and initiating early nucleational events necessary for matrix mineralization, including the transport of calcium (Ca^2+^) and inorganic phosphate (Pi). TEM revealed the presence of mineral granules within EVs secreted by MinOBs, particularly in larger vesicles ranging from 150 to 200 nm in diameter. (Figure 4A) SAED analysis indicated that these granules possess an amorphous structure (Figure 4B), and EDX spectroscopy confirmed the presence of both calcium and phosphorus elements. (Figure 4C) Previous studies have shown that Pi, generated through ALP activity, facilitates the transformation of amorphous calcium carbonate into amorphous calcium-phosphate (ACP), which in turn promotes hydroxyapatite deposition during osteoblast mineralization 16, 21, 32, 33. To further characterize the mineral phase, we compared the EV-associated granules with synthetic ACP using FTIR. The ν4 vibration peak of phosphate (PO₄^3-^) appeared near 580 cm^-1^, with a secondary band around 550 cm^-1^, consistent with the spectral signature of amorphous calcium phosphate 17. (Figure 4D) Additionally, a reduction in the asymmetric stretching vibration of amine groups (-NH₂) near 1400 cm^-1^ was observed, suggesting a decrease in membrane protein content and a shift in the ratio of large to small EVs. (Figure 4E)

Calcium phosphate deposits are are commonly found within mitochondria of mineralizing cells 34. Flow cytometric analysis demonstrated the presence of mitochondrial components in secreted EVs, (Figure 4F-G) suggesting that mitophagy in osteoblasts contributes to the formation of intramitochondrial ACP and its subsequent packaging into EVs. We observed a gradual increase in lysosome-mitochondria-mediated autophagy among the three OB subtypes. (Figure 4H) To assess mitochondrial function, we measured mitochondrial membrane potential (MMP) using TMRM staining and evaluated ATP production in EnOBs, StOBs, and MinOBs. Both polarization and energy metabolism were significantly elevated in StOBs (Figure S5A-B; Figure 4I) TEM imaging revealed distinct differences in ACP accumulation across OB subtypes. EnOBs displayed low-density mitochondrial vesicles, whereas StOBs contained numerous electron-dense granules. In MinOBs, high-electron-density granules were present, and some mitochondria appeared structurally compromised. EDX confirmed substantial calcium accumulation within MinOBs. (Figure 4J) These findings suggest that ACP interacts both with mitochondria and intracellular vesicles. During osteogenic progression, ACP with the same elemental composition was observed to accumulate within mitochondria and subsequently undergo degradation via mitophagy. These degraded materials were transferred to the extracellular matrix via EVs. The co-localization of ACP and collagen fibrils in the matrix signifies a key event marking the onset of mineralization 35. This transfer mechanism represents a critical step in bone formation and tissue calcification.

Altogether, these results demonstrate that EVs derived from different OB subtypes mediate distinct roles during osteogenesis and mineralization. In particular, MinOBs utilize mitophagy to transport ACP precursors from dysfunctional mitochondria into EVs. Concurrently, specific miRNAs within MinOB-derived EVs further facilitate mineral deposition. In the late stage of OA-related subchondral bone remodeling, both miRNA profiles and mineral granules contribute to enhanced osteogenic activity in BMSCs, driving the transition from bone resorption toward bone formation.

### Dynamic regulation of Wnt-mediated mitophagy in subchondral bone remodeling

**Figure 5:**
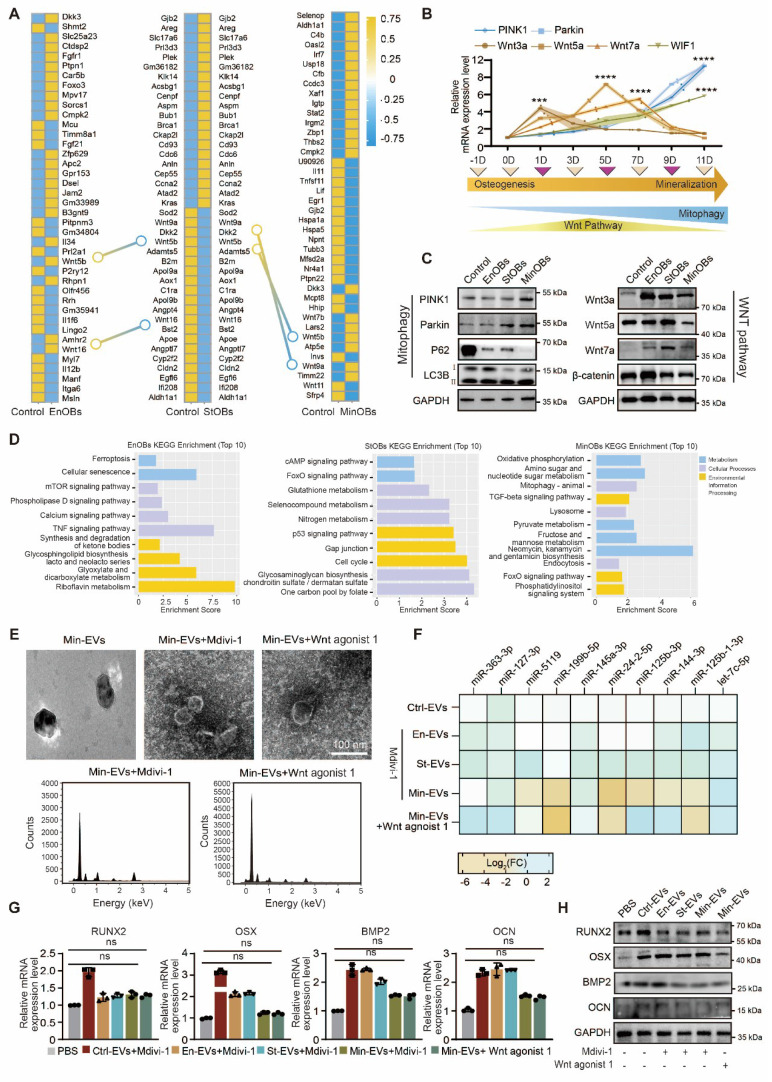
** Relationships between Wnt pathway and mitophagy in OB subtypes. (A)** Genes shown by heat map of top 40 DEGs in three OB subtypes.** (B-C)** Relative mRNA expression level and protein level of Wnt proteins and autophagy-related genes showed the dynamic changes in Wnt pathway and autophagy activity. **(D)** KEGG analysis of unique different expressed genes in OBs subtypes. **(E)** TEM images and EDX elemental mapping show no electron-dense granules in Mdivi-1 and Wnt agonist-1 treated-EVs. **(F)** miRNA expression level of 10 miRNAs in Mdivi-1 and Wnt agonist-1 treated Min-EVs (n = 3). **(G-H)** The expression levels of osteogenic maker genes in BMSC after treated with Mdivi-1 and Wnt agonist-1 treated-EVs.

Mitophagy in osteoblasts plays a crucial role in maintaining the balance of bone remodeling. A growing body of evidence suggests that impaired autophagy disrupts osteoblast maturation and perturbs the delicate equilibrium between bone formation and resorption 36. Recent studies further indicate that the regulatory role of autophagy in osteoblast differentiation and function is, at least in part, mediated through its interactions with osteogenic signaling pathways 37. In our analysis of differentially expressed genes (DEGs) among the three OB subtypes, we observed that the Wnt signaling pathway was upregulated in EnOBs and StOBs, but notably downregulated in MinOBs. Both RNA-seq results and previous experimental findings demonstrated that WIF1, a known Wnt pathway inhibitor, was highly expressed in MinOBs, further suggesting a shift in Wnt signaling activity. (Figure 5A, Figure S6C) Previous research has shown that Wnt inhibitors are upregulated during terminal stages of osteogenic differentiation 38. To explore the relationship between Wnt signaling and mitophagy, we next examined the expression of mitophagy-related genes. These markers were significantly upregulated in MinOBs. (Figure 5B-C) In addition, functional enrichment analysis based on subtype-specific genes revealed that EnOBs were associated with activation of the TNF signaling pathway, StOBs with the cAMP pathway, and MinOBs with increased mitophagy activity. (Figure 5D) While some genes were shared across all three subtypes (Figure S6A), these subtype-specific patterns pointed to distinct biological functions.

To further investigate the link between Wnt signaling and mitophagy, we treated OB subtypes with Mdivi-1, a selective mitophagy inhibitor. We then collected EVs under these conditions for further analysis. Mitochondrial markers COX4 and TOMM20, indicative of mitochondrial content, were reduced in EVs from cells treated with Mdivi-1 or Wnt agonist 1. (Figure S6B) Furthermore, RT-qPCR analysis showed a significant decrease in the expression of the 10 Min-EV-specific miRNAs following both treatments. Consistently, miRNA expression in EVs from Wnt agonist 1-treated MinOBs also declined. Alongside these changes, calcium and phosphate content within Min-EVs was diminished, as confirmed by TEM imaging and EDX analysis. (Figure 5E-F) Notably, inhibition of mitophagy signaling led to a reduced osteogenic potential in EVs derived from all three OB subtypes, as evidenced by decreased mRNA and protein levels of osteogenic markers. This reduction was most pronounced in Min-EVs. (Figure 5G-H) Moreover, ALP and ARS staining following Mdivi-1 or Wnt agonist 1 treatment revealed a marked decrease in staining intensity, further indicating impaired mineralization activity. (Figure S6C-D) Together, these findings suggest that alterations in Wnt signaling among the three OB subtypes are closely linked to changes in mitochondrial function, ranging from activation to autophagy. These mitochondrial dynamics appear to influence the osteogenic potential of EVs, which in turn may play a critical role in regulating the process of bone remodeling.

### OB subtypes-derived EVs increased osteoarthritic subchondral bone remodeling in DMM mice

**Figure 6:**
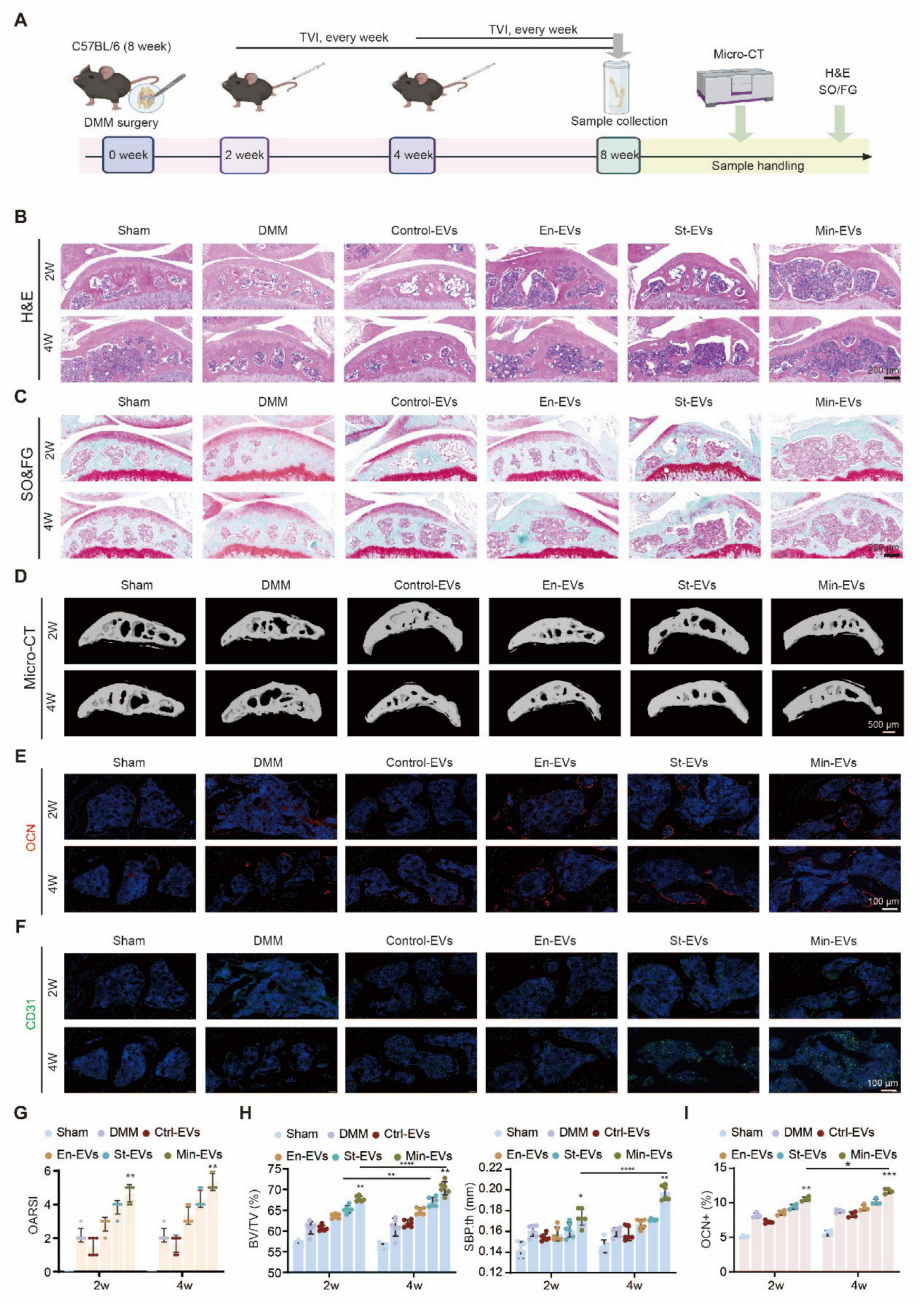
** EVs aggravated the subchondral bone remodeling in DMM mice at late administration stage. (A)** Schematic showing the strategy for DMM surgery and sample processing. EVs were injected into the tail vein every week after DMM surgery, and histological analysis was performed. H&E staining images **(B)**, and SO&FG staining **(C)** after 8 weeks of DMM modeling revealed distinct patterns (scale bar: 200 μm, n = 6 per group). **(D)** Representative 3D reconstruction of the medial tibial plateau subchondral bone sagittal plane**.** Scale bar: 500 μm. n = 6 per group.** (E)** Immunohistochemical staining of OCN (green) and CD31 (red).** (F)** in subchondral bone were quantified and presented. n = 4 per group. Scale bar: 100 μm.** (G)** Quantification of articular cartilage histological scores on the tibial plateau using OARSI criteria. n = 6 per experimental group.** (H)** Using Micro-CT analysis, subchondral bone parameters such as subchondral bone volume to total tissue volume (BV/TV) and subchondral bone plate thickness (SBP.th) were evaluated in the medial tibial plateau. n = 6 per group. **(I)** Quantification of OCN positive cells/mm^2^. n = 4 per group. Differences in mean values were evaluated for statistical significance using two-way ANOVA followed by Tukey's HSD multiple comparisons test at a significance level of p ≤ 0.05. The results, depicted graphically with representative images, are based on three independent replicates, and error bars represent the mean ± SD. Means sharing the same letters (ns) are not considered significantly different. The following significance levels were assigned: *p < 0.05, **p < 0.01, ***p < 0.001 and **** p < 0.0001. Quantitative data are presented as mean ± SD.

To investigate the role of EVs in OA progression, we initially administered EVs via tail vein injection at either 2 or 4 weeks following DMM surgery. (Figure 6A) Upon confirming the biosafety of the administered EVs, (Figure S7A) histological staining revealed structural damage in both articular cartilage and subchondral bone, which was exacerbated when EVs were delivered at the later time point. (Figure 6B-C) In contrast, the Ctrl-EVs group exhibited no apparent proteoglycan loss, and the OARSI scores of these joints remained comparable to those of the sham group. This finding is consistent with prior reports on the therapeutic effects of BMSC-derived EVs in OA models 39, 40. (Figure 6G) To evaluate temporal changes in subchondral bone architecture, we performed micro-CT analysis and 3D reconstruction at various time points following EV treatment. (Figure 6D) In the DMM-induced OA model, extensive osteophyte formation was observed by 8 weeks post-surgery. EV treatment resulted in a marked increase in subchondral bone mass, as evidenced by a bone volume to total volume (BV/TV) ratio of approximately 60-70%. Similarly, the subchondral bone plate thickness (SBP.th) was significantly elevated, (Figure 6H) indicating heightened bone remodeling activity. Immunostaining analyses further revealed that mice treated with Min-EVs exhibited significantly higher levels of OCN^+^ osteoblasts (Figure 6E, I) and CD31^+^ endothelial cells, (Figure 6F) compared to other groups. These findings suggest that Min-EVs promote osteoblast maturation and angiogenesis, thereby contributing to excessive mineralization and the development of subchondral sclerosis.

In a parallel experiment, EVs were administered via intra-articular injection to assess how articular cartilage modulates subchondral bone remodeling during OA progression. (Figure S9A) Notably, a mild reduction in proteoglycan content within cartilage was observed, particularly in the group receiving EVs at 2 weeks post-DMM surgery. (Figure S9B-C) This coincided with a significant increase in the OARSI score, suggesting early cartilage degeneration. (Figure S9G) Consistently, micro-CT analysis revealed that both BV/TV and SBP.th were elevated at the 2-week time point, indicating localized and uneven bone formation. (Figure S9D, H) Further analyses focused on type H vessels within the subchondral bone, which are known to support osteogenesis by coupling angiogenesis with new bone formation 23. Microangiographic analysis demonstrated that EV treatment significantly promoted neovascular infiltration into the subchondral region. (Figure S9E) Additionally, a pronounced increase in the OCN⁺ area was observed in the Min-EV group relative to other treatments, (Figure S9F, I) further supporting the notion that these vesicles enhance osteoblast activity. These results suggest that early-stage EV administration facilitates aberrant angiogenesis in the subchondral bone, which may accelerate cartilage erosion through vascular invasion.

Given the distinct pathological stages of OA initiation and progression 7, we next sought to determine whether the observed effects were dependent on the disease context. To this end, we injected the three types of EVs into the knee joints of healthy mice. (Figure S10A) While a notable increase in bone density was detected, (Figure S10B-C) neither cartilage degradation nor OARSI scores differed significantly from those in untreated controls. (Figure S10D-E) These results indicate that the pro-mineralization effects of osteoblast-derived EVs are amplified under OA-specific conditions.

Collectively, these findings highlight the stage- and context-dependent effects of OB-derived EVs on OA progression. Early administration of EVs may exacerbate vascular invasion and cartilage degeneration, whereas later intervention appears to promote subchondral sclerosis. This underscores the importance of identifying an optimal therapeutic window for EV-based interventions. Furthermore, the differential responses of cartilage and subchondral bone to EV treatment reflect their intrinsic heterogeneity in Wnt signaling and mitophagy activity, providing a rationale for the development of EV subtype-specific therapeutic strategies targeting osteochondral remodeling in OA.

## Discussion

The bone-cartilage interface operates as a functional unit, where subchondral bone and cartilage engage in dynamic molecular crosstalk 6. This interaction is influenced by the joint's physiological or pathological state and involves coordinated or disrupted signaling among osteocytes, osteoblasts, osteoclasts, endothelial cells, and sensory neurons within the subchondral bone niche 8. Effective osteoarthritis treatments require accurate assessment of joint tissue pathology at the time of therapy 41. Furthermore, alterations in subchondral bone are not uniform across different articulating joints in OA 42. Therefore, elucidating cellular heterogeneity is crucial for understanding the mechanisms underlying osteoarthritis. Building on our prior observation that osteoblast subpopulations derived from OA patients correlate with disease progression, this study aims to further delineate the distinct functions of these osteoblast subsets in subchondral bone remodeling throughout osteoarthritis development.

Nonetheless, the direct isolation of viable osteoblast subpopulations from the densely mineralized subchondral bone matrix at various osteoarthritis stages in both murine models and patients remains technically challenging. Therefore, we obtained osteoblast subtypes by inducing osteogenic differentiation of BMSC at different times in vitro 9. At the end of differentiation, we found the cell activity was largely decreased and secreted more crystallinity mineral deposition at 14 days, diminishing the ability to affect other cells 43. To comprehensively characterize the cellular states at different stages, we assessed the expression of established subtype marker genes and evaluated their basic osteogenic potential. In addition, we verified the functional characteristics of the three OB subtypes by analyzing their conditioned media, which revealed their distinct roles in promoting blood vessel invasion and chondrocyte degeneration. Specifically, EnOBs, representing the early differentiation stage, were associated with bone-vascular coupling and early bone formation. StOBs, corresponding to an intermediate stage, exhibited high expression levels of genes related to extracellular matrix remodeling and bone fibrogenesis, suggesting their potential involvement in OA-associated fibrosis. MinOBs, as a terminally differentiated subpopulation, specifically express mature bone markers and show significant upregulation of the Wnt signaling antagonist WIF1. Previous research has demonstrated that the Wnt/β-catenin signaling pathway contributes to bone matrix regulation through the upregulation of miR-29b. Furthermore, inhibition of Wnt signaling has been shown to promote PINK1/Parkin-mediated mitophagy, indicating that Wnt may influence cellular metabolic balance by modulating both miRNA expression and mitochondrial autophagy, thereby establishing an interconnected regulatory network 44, 45. This suggests their critical role in matrix mineralization and pathological osteosclerosis.

At the end stage of osteoarthritis, increased bone formation results in the densification of the subchondral plate. And in our previous study, we also found the proportion of mineral osteoblast increased in subchondral bone at 8 weeks after DMM surgy mice 19. Consequently, structural deterioration in the subchondral bone during OA is closely associated with osteoblast-driven bone formation, particularly involving mature osteoblasts. Study showed that osteoblast-derived EVs are a heterogeneous population, mediating cell-cell communications and regulate diverse cell phenotype in bone disease 46, 47. As the complex structure in bone-cartilage unit, EVs with its flexible particle size are the preference to deliver biochemical and molecular information. Uenaka, M.* et al.* proposed that extracellular vesicles derived from mature osteoblasts can induce a shift from bone formation toward bone resorption *in vivo* 48. Wu, X. *et al.* suggested that exosomes specifically originated from the subchondral bone, carry a microRNA known as miR-210-5p, which further accelerating subchondral bone remodeling 12. However, our findings suggested that the identified three OB subtypes seems to promote osteogenic of BMSCs, which supported by the findings of another reported study 9, 47. With inhibitor of EVs administration, we further confirmed the different osteogenic potential of three EVs plays an important role in the bone formation.

Wei, Y. *et al.* merely observed the different osteogenic potential of three EVs, we also demonstrated that the difference depends on the contents in EVs. We analyzed miRNAs in three EVs for the first time via next-generation sequencing. We found 34 intersection miRNAs in three EVs and their target pathways enrichment analysis suggested the basic function of bone matrix formation. Here, we mainly focused on the difference in Min-EVs, because stronger capacity of osteogenic potential and closely related to bone sclerosis in late-stage OA. Obviously, unique 10 miRNAs targeted mineral deposition, which is the necessary condition for further stage of bone formation 49. These unique miRNAs largely related to the function of its origin cells, which partial gave a reason to the stronger capacity of osteogenic potential. On the other hand, Wei, Y. *et al.* observed a gradual increase in the proportion of MVs (matrix vesicles) and small-sized EVs during the osteoblasts differentiation progression 9. Here, we also found Min-EVs with two peaks associated with different sizes: one around 50 nm in diameter (similar to normal EVs) and one around 200 nm in diameter (similar to MVs), which supported the study. After the penetrating analysis of MinOBs, we verified the transport pathway of ACP into ECM, which was put forward before 34. Thus, the experimental results not only elucidated the unique mineralization function of Min-EVs, but emphasize three EVs inherit the different function of three OB subtypes. Based on these results, our ongoing research is expanding to investigate the contributions of osteoclasts and the immune microenvironment to osteoarthritis pathogenesis. Emerging evidence suggests that osteoblast-derived EVs may regulate osteoclastogenesis either directly by delivering specific miRNAs or proteins or indirectly by modulating immune cell functions. Based on these insights, we hypothesize that osteoblast-derived EVs may not only directly influence osteoclast differentiation but also modulate the OA progression through immunoregulatory mechanisms. Our preliminary experiments have demonstrated that Min-EVs can suppress osteoclast differentiation, and further investigations into the underlying mechanisms and functional optimization are currently underway. In future work, we aim to systematically dissect the multi-target regulatory roles of OB-derived EVs within the bone-cartilage-immune interaction network, offering new perspectives for OA therapy.

The observation of ACP leads us to notice the mitophagy in osteogenic differentiation. The role of mitochondria during the process of osteogenesis has been suggested 37, but the cause of alternation state of mitochondria with time is not clear. WIF1, recognized as a distinctive marker of MinOBs, exhibits elevated expression levels during the late phase of osteogenic differentiation 50. Study showed that under chronic inflammatory conditions, activation of the Wnt/β-catenin pathway was found to inhibit mitophagy, impairing the differentiation of MSCs 28. Additionally, Wnt signaling has been highlighted as a potential therapeutic target for OA, further prompting us to examine its role in osteoblast heterogeneity. Our findings revealed that Wnt signaling exhibits dynamic changes throughout OB differentiation, closely correlating with functional heterogeneity. During the EnOB and StOB stages, Wnt activity was elevated, promoting osteogenic gene expression, angiogenesis, and maintaining mitochondrial function—all of which contribute to balanced bone remodeling. However, at the mineralizing stage, Wnt signaling was markedly downregulated, while WIF1 expression was upregulated. This shift was accompanied by mitochondrial dysfunction, increased mitophagy, and ACP accumulation, ultimately facilitating EV-mediated pathological mineralization. These alterations highlight a critical mechanism underlying subchondral bone plate thickening and sclerosis in late-stage OA. Based on these findings, we propose a stage-specific intervention strategy targeting the "Wnt-mitophagy axis." In early-stage OA, moderate activation of Wnt signaling could help maintain bone homeostasis while avoiding premature mineralization. In contrast, during late-stage OA, regulating Wnt activity or inhibiting Drp1-dependent mitophagy may effectively block pathological mineralization cascades. It is crucial to carefully define the therapeutic window—excessive Wnt activation may induce ectopic ossification or tumorigenesis, while excessive inhibition could impair bone repair. Similarly, disrupting mitophagy balance could lead to metabolic dysfunction or systemic toxicity. In summary, the Wnt-mitophagy-ACP-EV axis identified in this study not only provides a novel mechanistic explanation for subchondral bone sclerosis in late-stage OA but also offers a theoretical basis for exploring stage-specific intervention strategies. These findings further support the role of Wnt signaling as a regulator of mitophagy in modulating osteogenic differentiation.

The *in vivo* roles of OB-derived EVs in OA pathology remain largely unexplored. To more accurately replicate the pathological milieu of osteoarthritis, we utilized an integrated approach combining *in vitro* mechanistic studies with *in vivo* validation in the DMM mouse model.* In vitro* experiments focused on evaluating the direct effects of EVs on bone-cartilage interface metabolism under normal physiological conditions, without introducing inflammatory stimuli such as IL-1β. This approach was chosen to avoid masking the specific regulatory effects of EVs on matrix homeostasis, as inflammation can independently induce matrix degradation. Preliminary results indicated that EVs could significantly suppress matrix degradation pathways, such as MMP13 activation, under non-inflammatory conditions, but exhibited limited anti-inflammatory effects when applied in an inflammatory microenvironment. *In vivo* experiments further demonstrated the subtype-dependent functions of OB-derived EVs. Specifically, MinOB-EVs accelerated OA progression by promoting subchondral bone sclerosis, whereas EnOB-EVs exerted bone-protective effects. This integrated *in vitro* and *in vivo* analysis suggests that EVs may influence OA-related remodeling primarily through non-inflammation-dependent metabolic reprogramming mechanisms, with their effects being modulated by the stage-specific microenvironment of OA.

**Figure 7:**
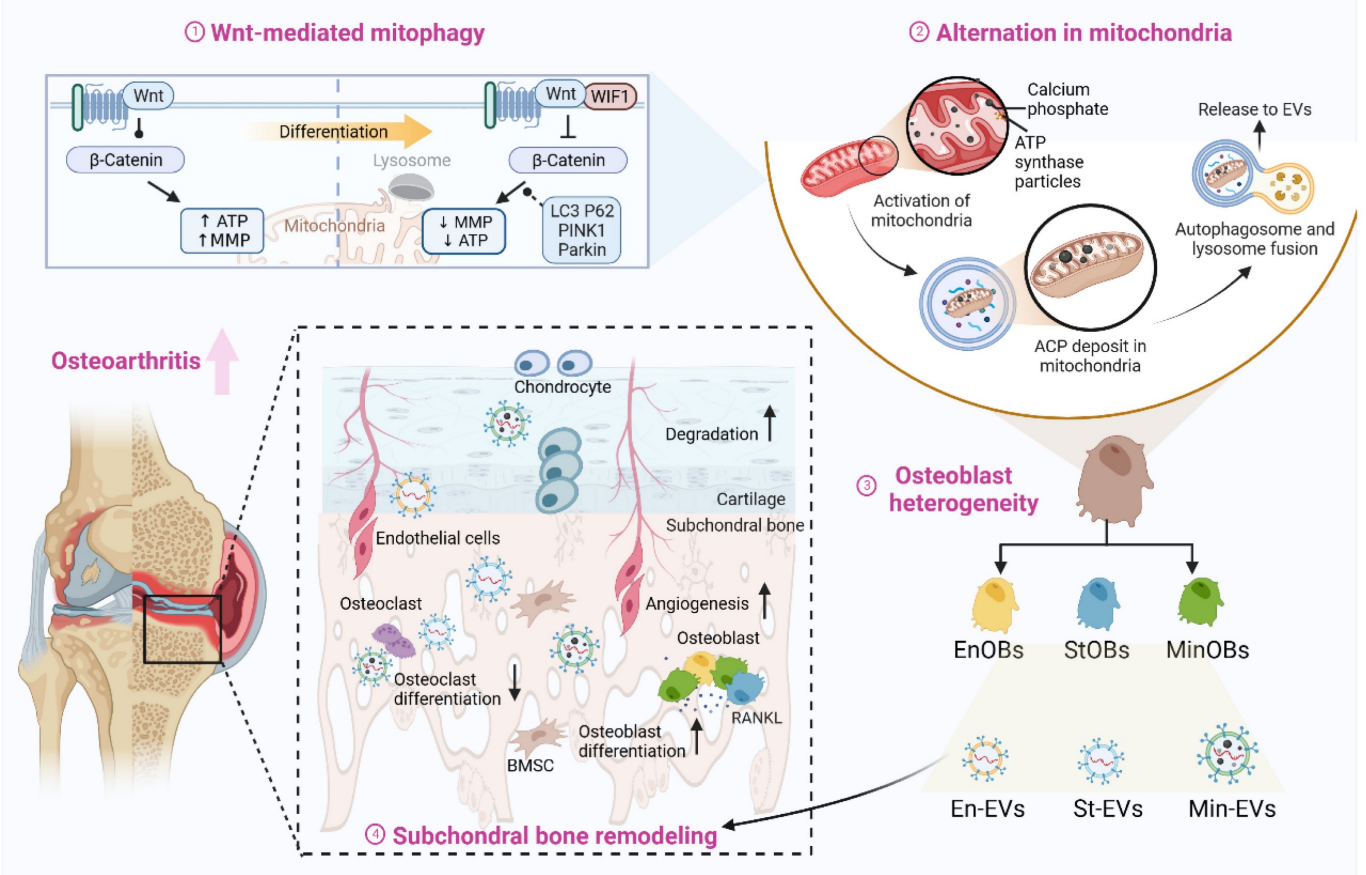
** Osteoblasts subtye-derived EVs aggravated subchondral bone remodeling in OA progression.** Regulated by Wnt pathway, osteoblasts were heterogeneous in osteogenic differentiation and the activation of mitophagy will provide calcium phosphate for further mineralization. EVs from three OB subtypes inheriting the heterogeneity could promote osteogenic with functional miRNAs and calcium phosphate. Osteoblasts subtypes-derived EVs could accelerate the subchondral bone remodeling in OA progression, resulting in bone sclerosis and cartilage degeneration. Created by Biorender.

Using the DMM model, we tested two administration strategies: systemic injection (primarily targeting subchondral bone) and local intra-articular injection (primarily affecting cartilage). Considering the limited bone-targeting ability of the three EV types 9, the result of systemic injection still suggested early administration could inhibit the osteoclast-mediated bone resorption at early-stage OA. However, since the microstructure change in OA subchondral bone was formed at 4 weeks after DMM, promoting osteogenic differentiation of BMSC will continues to deteriorate the bone formation, leading to osteophyte and osteosclerosis. Another administration method showed different results. Early intra-articular injection destroyed the chondrocyte matrix by miRNAs and calcium phosphorus 51, coupled with the angiogenesis capacity of three EVs. This, in turn, enhanced the crosstalk between ECs and chondrocytes, thereby promoting bone resorption and enabling the infiltration of oxygen-rich type H vessels into the subchondral bone 18. During the late stages of OA, cartilage degradation leads to increased porosity within the subchondral bone plate, resulting in enhanced uptake of EVs 8. Moreover, Wnt signaling within both cartilage and subchondral bone is critically involved in the pathogenesis of osteoarthritis, exerting effects through both independent and synergistic mechanisms 52, 53. Three EVs maintained the different levels of Wnt activation from three OBs subtypes, which also explained Wnt-related therapeutics targeting different osteoarthritic tissue will exhibit complex outcome 2. Therefore, the timing of OA intervention is critical. Administration of anti-osteoclast agents should be initiated during the early stages of OA to effectively delay disease progression. Conversely, at the developing or advanced stages of OA, such interventions—particularly those that promote osteogenic differentiation—may yield paradoxical effects and potentially exacerbate pathological bone remodeling. Although direct therapeutic effects of EVs were not observed, our results strongly indicate that EVs from specific osteoblast subtypes actively regulate subchondral bone remodeling in advanced osteoarthritis. (Figure 7)

At present, no biomarkers have been established for the early identification of osteoarthritis patients characterized by accelerated subchondral bone remodeling 2. Currently available disease-modifying OA drugs (DMOADs) targeting subchondral bone remodeling represent disappointing results, such as bisphosphonates (limiting bone resorption by targeting osteoclasts), Cathepsin K (a protease produced mostly by osteoclasts), and MIV-711 (limit cartilage damage and bone remodeling). In spite of these efforts have not acquire optimistic returns, our study made an explanation for the appropriate intervention time targeting bone remodeling is important in OA therapy. On the other hand, the biomarkers of osteoblast could be an index to identified the late-stage of OA, which indicating the degree and stage of subchondral bone remodeling.

Some limitations of this study should be noted. First, the osteoblast subtypes were derived from normal physiological conditions *in vitro*. It is better to make efforts to acquire from OA mice in pathological state, thus, the disease-stage is stronger relate to the osteoblast phenotype. Second, the specific role of miRNAs in three EVs could be further investigated to explore OA-related signaling pathway. Besides, the specific relationship between Wnt pathway and mitophagy should be further investigated. Last, three EVs showed the osteogenic potential, and the engineered EVs demonstrated an ability to remain within the bone matrix for an extended period. Thus, we are developing engineered Min-EVs targeting inhibiting bone resorption and promoting bone formation as a therapeutic method.

In conclusion, we have firstly reported the mechanism of heterogeneous OB subtypes during osteogenic progress was regulated by Wnt pathway. We also verified functional EVs with miRNAs and ACP generated from them are affected by the activation of mitophagy. The therapeutic window to intervene OA bone remodeling should consider the dynamic process of OA progression.

## Supplementary Material

Supplementary figures.

## Figures and Tables

**Table 1:** The potential function of 10 miRNAs in Min-EVs.

miRNA	Role	Reference
miR-let-7c-5p	Reduced proinflammatory cytokine production	54
miR-125b-1-3p	Targeting Bcl-2-mediated apoptosis	55
miR-144-3p	Inhibiting chondrocyte apoptosis and inflammation by the MAPK signaling pathway	56
miR-125b-3p	Negative regulator of early chondrogenic differentiation	57
miR-24-2-5p	Higher in osteoporotic fractures	58
miR-145a-3p	Mediate distinct pathogenic mechanisms in dietary-induced metabolic disorders	59
miR-199b-5p	Inhibited osteogenic differentiation; mechano-responsive pathways in OA chondrocyte	60,61
miR-5119	Resulting in robust anti-tumor cell immune response, upregulated cytokine production	62
miR-127-3p	Blocking the Wnt/β-catenin pathway activation	63
miR-363-3p	Promote chondrocyte injury and apoptosis; promotes BMSC osteogenic differentiation	64,65
