# Supplementary material for: Bone remodeling stimulated by Wnt-mediated mitophagy regulated extracellular vesicles in subchondral bone contributes to osteoarthritis development
Source: Theranostics. 2025 Sep 21;15(18):10007–27. doi: 10.7150/thno.111724 (PMC12486141; doi:10.7150/thno.111724)
Supplement: Supplementary file 1 — Supplementary figures. [file thnov15p10007s1.pdf]

Supplementary Materials for

Bone remodeling stimulated by Wnt-mediated mitophagy regulated extracellular vesicles in subchondral bone contributes to osteoarthritis development

Yuyuan Gu, Qirong Zhou, Shihao Sheng, Huijian Yang et al.

*Corresponding author. Chenglong Wang (wangorth@163.com), Haobo Tan (tantoo@163.com), Ke Xu (kexu@shu.edu.cn) or Jiacan Su (drsujiacan@163.com).

This PDF file includes:
Figs. S1 to S10

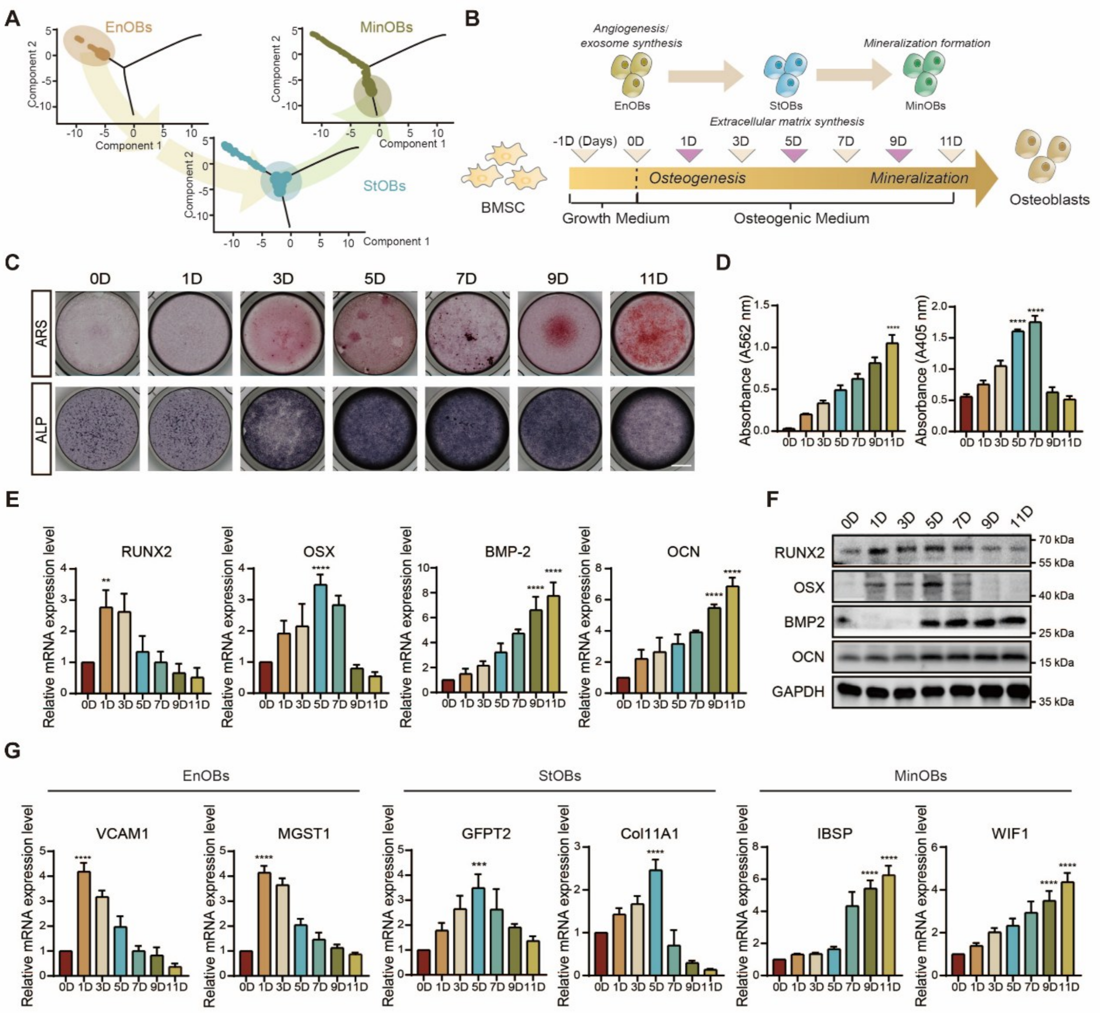

Figure S1. Osteogenic induction in BMSC triggers dynamic changes in osteogenic functions.

(A) The trajectory analysis of three osteoblasts subtypes in subchondral bone from OA patients. **(B)** Overview of the method for obtaining of three OBs subtypes. **(C-D)** Alizarin red S (ARS) and alkaline phosphatase (ALP) staining with relative quantitative analysis in the osteogenic process. Scale bar: 200 μm. **(E-F)** qRT-PCR and Western blot analysis of osteogenic markers (RUNX2, Osterix, BMP-2, OCN). **(G)** qRT-PCR demonstrating expression levels of specific genes marking EnOBs, StOBs, and MinOBs. VCAM1 (Vascular Cell Adhesion Molecule-1): a classic angiogenesis-related molecule that promotes the early vascularization process of osteogenesis by mediating the adhesion between EnOBs and vascular endothelial cells.

MGST1 (Microsomal Glutathione S-Transferase 1): highly expressed in EnOBs, which may be related to the regulation of oxidative stress response and cell protection function. COL1A1 (Collagen Type I Alpha 1 Chain): a classic matrix production marker that reflects the enhanced collagen fiber assembly ability during the transformation of cells to structural OBs. GFPT2 (Glutamine–Fructose-6-Phosphate Transaminase 2): related to matrix synthesis and metabolic reprogramming, and may be involved in the metabolic adaptation process of StOBs in tissue repair. IBSP (Integrin Binding Sialoprotein): an important non-collagen protein in bone matrix, accounting for about 12% of the non-collagen content of bone, plays an important role in bone calcification, and is commonly found in terminally differentiated OBs. WIF1 (Wnt Inhibitory Factor 1): an antagonist of the Wnt/β-catenin pathway, significantly upregulated in MinOBs, which can inhibit cell proliferation and promote mineralization, and is an important marker of its terminal differentiation state.

Differences in mean values were evaluated for statistical significance using Tukey's HSD multiple comparisons test at a significance level of $p \leq 0.05$. The results, depicted graphically with representative images, are based on three independent replicates, and error bars represent the mean ± SD. Means sharing the same letters (ns) are not considered significantly different. The following significance levels were assigned: *$p < 0.05$, **$p < 0.01$, ***$p < 0.001$ and ****$p < 0.0001$. Quantitative data are presented as mean ± SD.

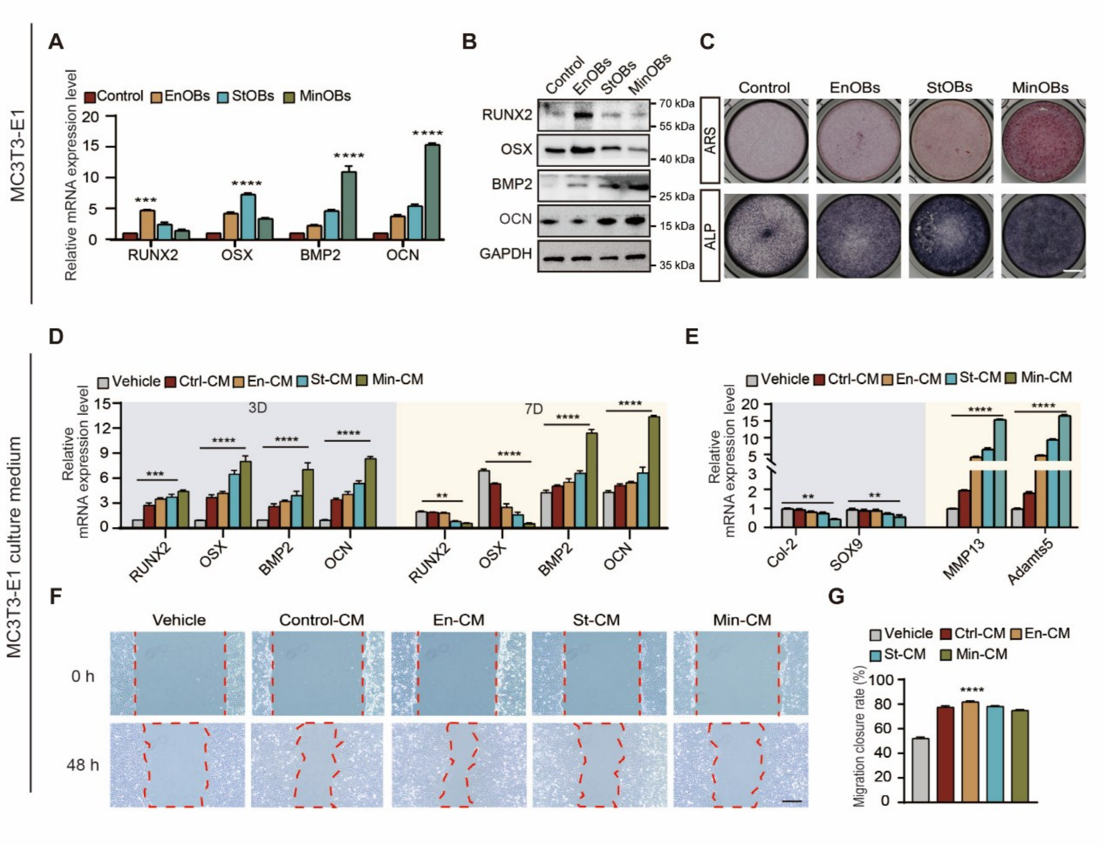

Figure S2. Functions of OB subclusters originated from MC3T3-E1.

(A-B) Western blot and qRT-PCR analysis of osteogenic genes in three OB subtypes from MC3T3-E1. **(C)** ARS and ALP staining of three OB subtypes. Scale bar: 200 µm. **(D)** qRT-PCR analysis of osteogenic gene expression in BMSC with conditioned medium (CM) from MC3T3-E1. **(E)** qRT-PCR analysis of SOX9, Col-2, MMP13, and Adamts5 in chondrocytes treated with MC3T3-E1 CM. **(F)** Wound healing examination of migratory capacity in HUVECs after treatment with MC3T3-E1 CM for 48 h. Scale bar: 500 µm. **(G)** Statistical analysis of HUVEC migration rate.

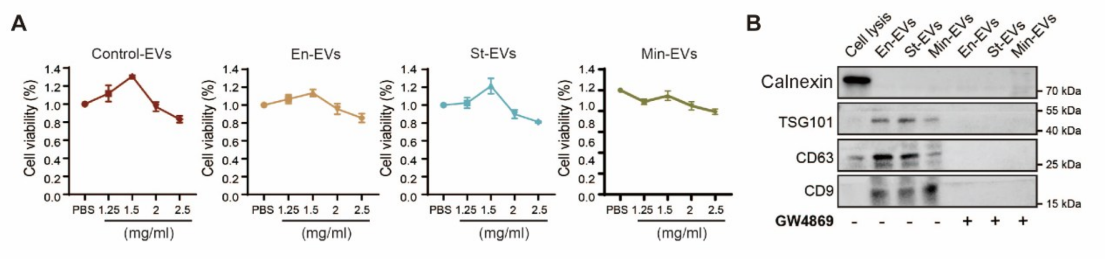

Figure S3. Functionalization of EVs.

(A) Cell viability of BMSC treated with EVs. **(B)** Expression of the marker proteins in GW4869-treated EVs.

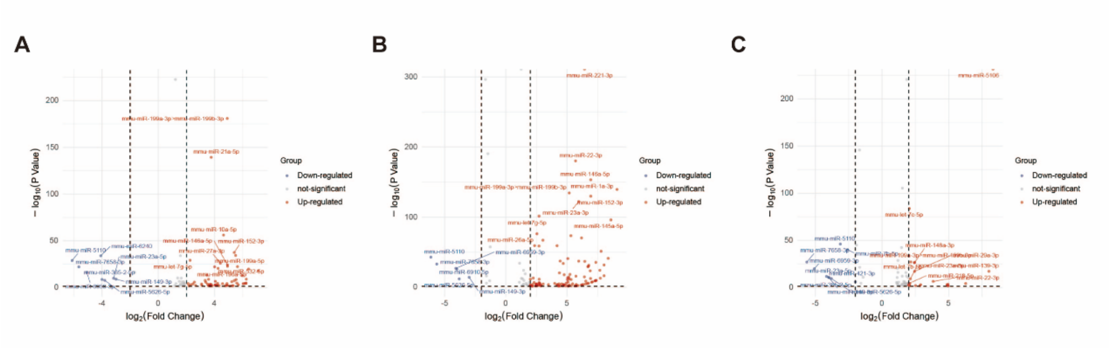

Figure S4. Differentially expressed miRNAs in EVs.

(A) Volcano plot of differentially expressed miRNAs in En-EVs. **(B)** Volcano plot of differentially expressed miRNAs in St-EVs. **(C)** Volcano plot of differentially expressed miRNAs in Min-EVs.

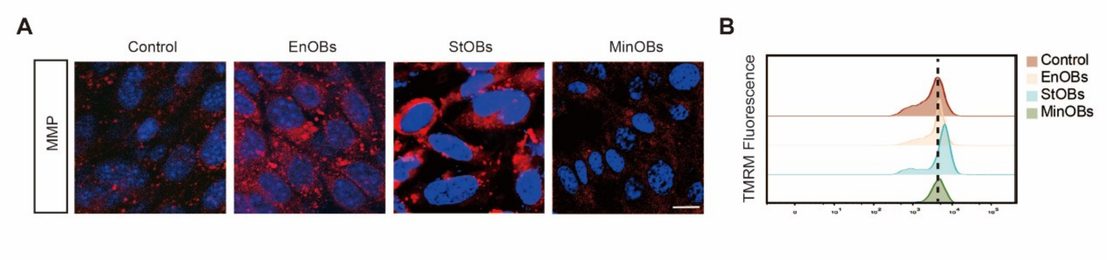

Figure S5. Mitochondria membrane potential (MMP) in OBs sublusters.

(A) OB subtypes were cotreated with TMRM (10 μm) for 15 min and then analyzed by flow cytometry **(B)** to quantify membrane potential (n = 3). Two-tailed Student's t-test. Scale bar: 10 μm.

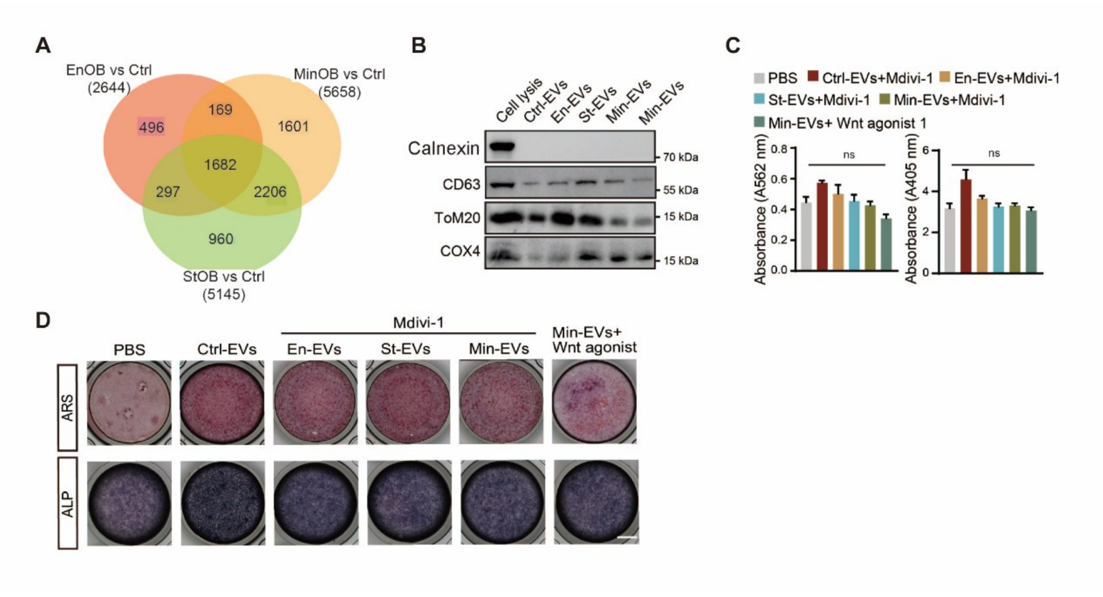

Figure S6. The osteogenic function was inhibited by Mdivi-1 and Wnt agonist-1.

(A) Venn analysis between differentially expressed genes in three OBs subtypes. **(B)** The mitochondria proteins were reduced in Mdivi-1 or Wnt agonist-1 treated EVs. **(C-D)** ARS and ALP staining of BMSC with Wnt agonist-1 treated EVs for 5 days and 9 days respectively and relative quantitative analysis. Scare bar: 500 µm.

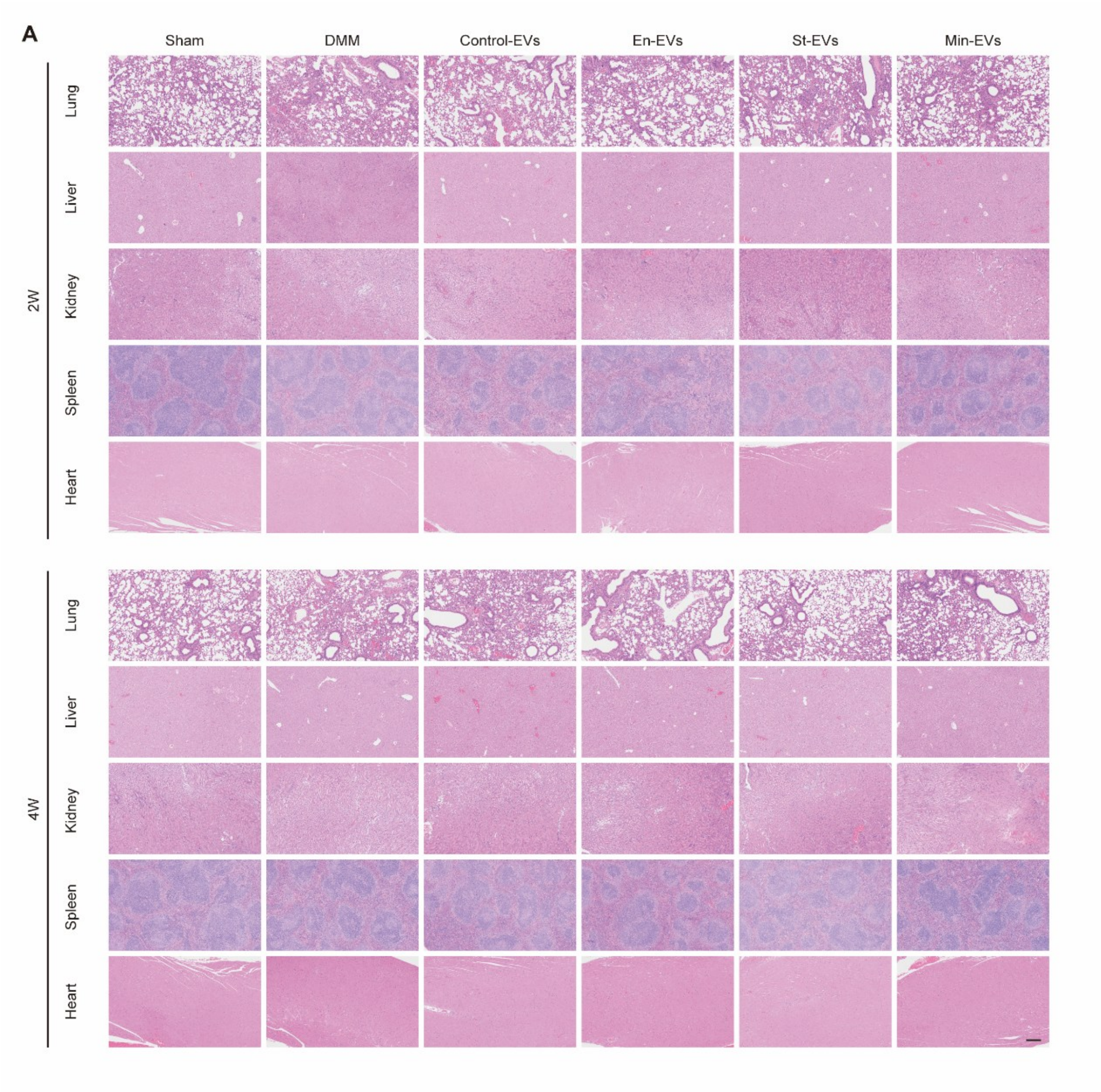

Figure S7. Systemic safety assessment of EVs.

(A) H&E staining of heart, liver, spleen, lung and kidney from mice showed no nontoxic of EVs. Scare bar:200 μm. n = 4 per group.

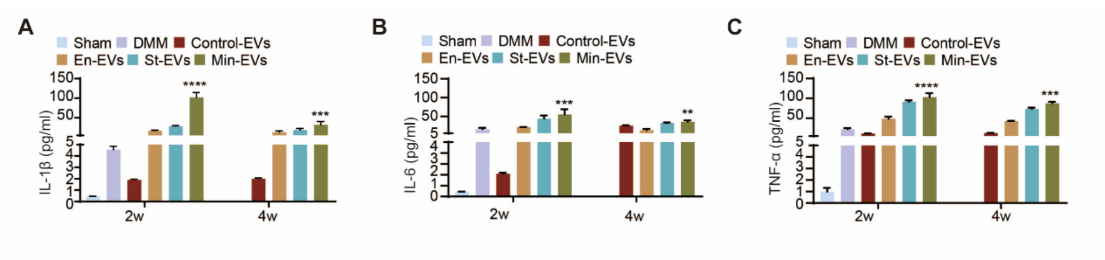

Figure S8. Inflammatory factors in serum.

(A-C) The concentrations of IL-1β, IL-6, and TNF-α in serum samples. n = 3 per group.

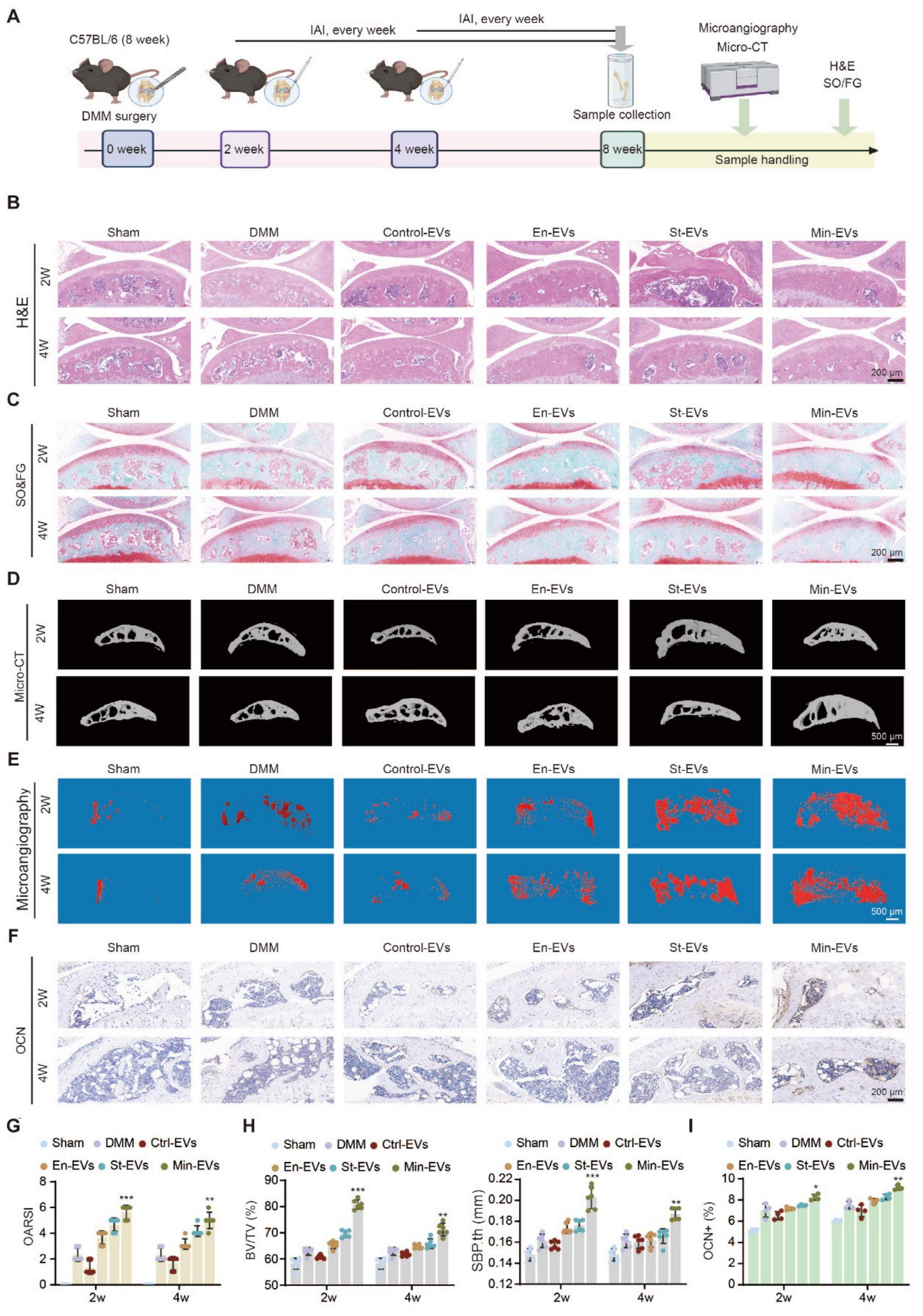
A
C57BL/6 (8 week)
IAI, every week
IAI, every week
Microangiography
Micro-CT
H&E
SO/FG
DMM surgery
Sample collection
0 week
2 week
4 week
8 week
Sample handling
B
Sham
DMM
Control-EVs
En-EVs
St-EVs
Min-EVs
H&E
2W
4W
200 μm
C
SO&FG
D
Micro-CT
500 μm
E
Microangiography
F
OCN
G
OARSI
H
BV/TV (%)
SBP.th (mm)
I
OCN+ (%)
Sham
DMM
Ctrl-EVs
En-EVs
St-EVs
Min-EVs
2w
4w
***
**
*

Figure S9. EVs aggravated the cartilage degeneration in DMM mice at early-stage administration.

(A) The diagram illustrates the methodology used for destabilization of DMM surgery and the subsequent steps involved in processing the samples. **(B)** Histological examination using H&E staining of the EVs administered each week following the DMM surgery. Images depict the stained samples. Scale bar: 200 μm. n = 6 per group. **(C)** Staining of knee joint sections with SO&FG conducted 8 weeks post-DMM surgery. Scale bar: 200 μm. n = 6 per group. **(D)** 3D reconstructed images of the sagittal plane of the medial tibial plateau's subchondral bone, captured 8 weeks after the DMM procedure. Scale bar: 500 μm. n = 6 per group. **(E)** 3D reconstructions displaying the sagittal plane of the medial subchondral bone blood vessels within the tibia, as visualized by Micro-CT. Scale bar: 500 μm. n = 6 per group. **(F)** Representative images of immunohistochemical staining for Osteocalcin in the subchondral bone. Scale bar: 200 μm. n = 4 per group. **(G)** Evaluation of the articular cartilage in the tibial plateau using the OARSI histological scoring system. n = 6 per group. **(H)** Quantitative analysis of the subchondral bone volume to total tissue volume (BV/TV) ratio and the measurement of subchondral bone plate thickness (SBP.th) in the medial tibial plateau, conducted using Micro-CT. n = 6 per group. **(I)** Quantitative assessment of the density of OCN-positive cells per square millimeter. n = 4 per group.

Differences in mean values were evaluated for statistical significance using two-way ANOVA followed by Tukey's HSD multiple comparisons test at a significance level of $p \leq 0.05$. The results, depicted graphically with representative images, are based on three independent replicates, and error bars represent the mean ± SD. Means sharing the same letters (ns) are not considered significantly different. The following significance levels were assigned: *$p < 0.05$, **$p < 0.01$, ***$p < 0.001$ and **** $p < 0.0001$. Quantitative data are presented as mean ± SD.

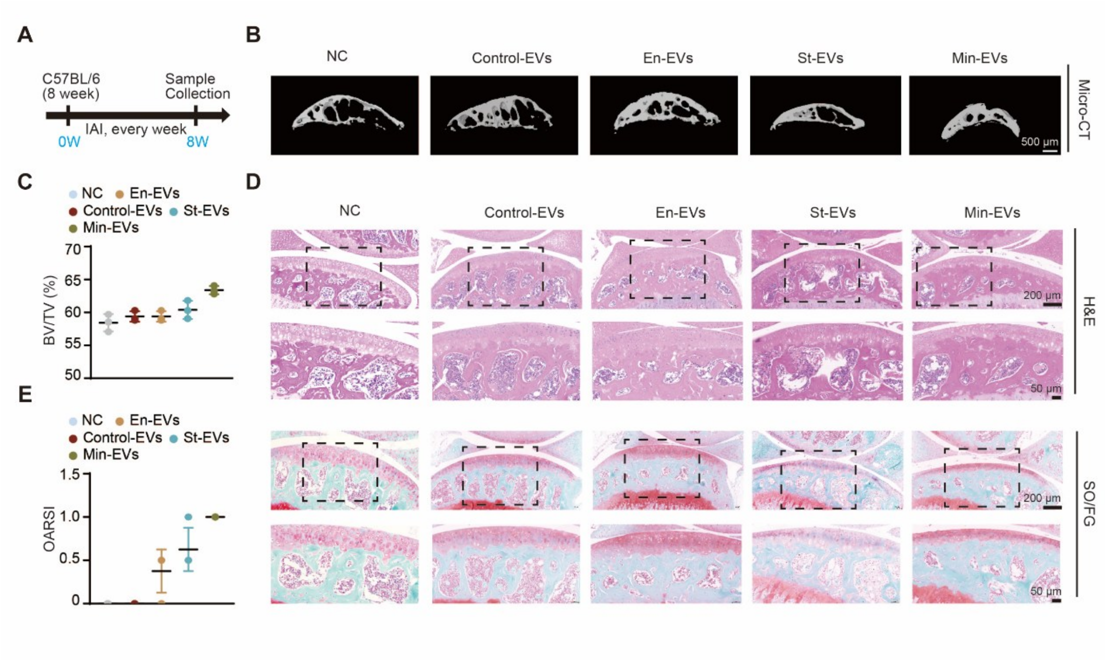

Figure S10. EVs have no effect on the induction of OA.

(A) This diagram outlines the protocol for administering EVs in healthy mice, detailing the method and timeline of administration. **(B)** 3D reconstructed images showing the sagittal plane of the medial tibial plateau's subchondral bone, taken 8 weeks post-administration. n = 3 per group. **(C)** This represents the numerical analysis of the subchondral bone volume to total tissue volume (BV/TV) ratio, providing insights into bone density changes. n = 3 per group. **(D)** H&E staining images of tissue samples from healthy mice receiving weekly intraperitoneal injections of EVs. Each experimental group consisted of three mice. Scale bar: 200 μm (first line). Scale bar: 100 μm (second line). n = 3 per group. And images of knee joint sections stained with SO&FG conducted 8 weeks after the commencement of EVs administration. Scale bar, 200 μm (first line). Scale bar, 50 μm (second line). n = 3 per group. **(E)** OARSI scoring of the articular cartilage in the tibial plateau, utilized to evaluate the impact of EVs on cartilage health. n = 3 per group.
